# Deciphering Anti‐Cancer Drug Efficacy Through Nanomechanical Vibrations in Living Gastric Cancer Organoids

**DOI:** 10.1002/advs.76878

**Published:** 2026-08-11

**Authors:** Ting Zhang, Qiubo Chen, Shihai Lan, Liang Zhu, Shuaiyu Liu, Yan Xia, Yalu Zhang, Hanhui Yao, Yongman Liu, Shangquan Wu, Qingchuan Zhang

**Affiliations:** ^1^ Department of Modern Mechanics CAS Key Laboratory of Mechanical Behavior and Design of Material University of Science and Technology of China Hefei China; ^2^ Department of Gastric Surgery Division of Life Sciences and Medicine The First Affiliated Hospital of USTC University of Science and Technology of China Hefei China; ^3^ MOE Key Laboratory of Cellular Dynamics CAS Center for Excellence in Molecular Cell Science University of Science and Technology of China Hefei China; ^4^ Beijing University of Chinese Medicine Third Affiliated Hospital Beijing China; ^5^ School of Biomedical Engineering Anhui Medical University Hefei China

**Keywords:** biomechanics, deep learning, drug screening, nanomechanical vibration, organoids mechanical model, patient‐derived gastric organoids

## Abstract

Cancer poses a severe threat to human health. The limited sensitivity and efficiency of conventional drug screening methods create a pressing need for novel anti‐cancer drug screening platforms. This study developed a drug efficacy assessment platform that integrates patient‐derived gastric cancer organoids, atomic force microscopy (AFM)‐based nanomechanical vibration detection, deep learning analysis and an organoid mechanical model. The platform enables non‐destructive detection of intrinsic nano‐vibrations of organoids, revealing that their amplitude and spectral characteristics are sensitive to the cytoskeleton, cell‐cell junctions, and cellular metabolism. Through the optimization of a deep learning model, we achieved high‐precision classification of organoids under pharmacological perturbation. The classification requires only 0.1 s of vibration data and achieves an overall accuracy of 97%. Applied to gastric cancer organoids, our platform detected the effects of picomolar (pM) concentrations of paclitaxel, notably preceding any observable morphological changes or apoptosis signals. Furthermore, the organoid mechanical model accurately predicted drug‐induced alterations in both the nanomechanical vibration amplitude and spectrum. These findings validate the platform's advantage for the highly sensitive, non‐destructive, and efficient screening of anti‐cancer drugs. Collectively, this work establishes a paradigm for organoid‐based drug evaluation through dynamic biophysical profiling and holds promise for advancing personalized cancer treatment strategies.

## Graphical abstract

This study developed an efficacy assessment platform that integrates patient‐derived gastric cancer organoids, atomic force microscopy (AFM)‐based nanomechanical vibration detection, deep learning analysis, and organoid mechanical modeling. It detects picomolar drug effects within 0.1 s signal, achieves 97% classification accuracy, and offers non‐destructive, high‐sensitivity drug screening for personalized cancer therapy.

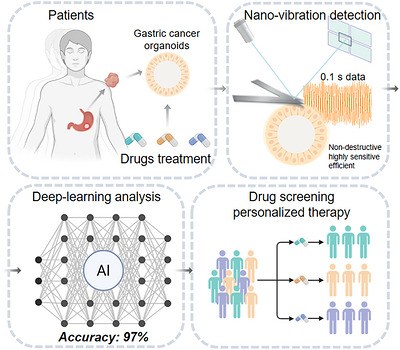

## 1 Introduction

Cancer poses a significant threat to global health. Among various malignancies, gastric cancer remains one of the most devastating worldwide, with persistently high incidence and mortality rates [1, 2]. Despite advances in surgical techniques, chemotherapy, targeted therapy, and immunotherapy, the overall prognosis for gastric cancer patients is still unsatisfactory, and individual responses to treatment vary dramatically [3]. Therefore, there is an urgent need to develop new platforms for the individualized drug efficacy assessment. However, traditional two‐dimensional (2D) cell culture models lack tissue architecture and cellular heterogeneity, making them poor predictors of drug efficacy. Moreover, animal models are limited by species differences, high costs, and low throughput, failing to meet the urgent need for rapid and precise drug response evaluation in personalized medicine [4]. Recent breakthroughs in organoid technology have provided a revolutionary solution to this dilemma. Organoids are miniature organ analogues that form through the self‐organization of stem cells or tissue‐derived progenitor cells under three‐dimensional (3D) culture conditions. They can partly recapitulate the complex structure, diverse cell types, and key physiological functions of their source tissue [5, 6, 7, 8]. Patient‐derived organoids (PDOs) faithfully retain the genomic features, histopathological architecture, and heterogeneous drug response of the original tumor. They are widely recognized as an ideal platform for disease modeling, mechanistic studies, and individualized drug screening [9, 10, 11, 12].

However, the current paradigm for organoid‐based drug screening remains tethered to traditional cell pharmacology methods, primarily relying on endpoint measurements such as cell viability (e.g., ATP content), morphological disintegration, changes in specific molecular marker expression, and genomic or transcriptomic sequencing [13, 14]. These methods typically have drawbacks and limitations, including low sensitivity (e.g., insensitivity to subtle phenotypic changes), non‐quantitative outputs (e.g., qualitative, or semi‐quantitative scoring of morphology), time‐consuming (e.g., endpoint assays requiring days to a week for results), invasiveness (e.g., destructive operations such as staining and fixation), and high cost (e.g., reliance on expensive assays like genome sequencing). Therefore, developing a detection technology capable of highly sensitive, efficient, and non‐destructive monitoring of functional state changes in organoids under drug treatment is crucial for deepening our understanding of drug mechanisms, enabling accurate pharmacodynamic assessment.

Living systems are not merely collections of biochemical reactions but are highly dynamic physical entities [15, 16]. The mechanical properties of cells and tissues—including their stiffness, intrinsic subtle movement—are intimately linked to their physiological state and pathological alterations [17, 18]. For instance, our previous work, together with studies from other groups, has successfully used nanomechanical sensors to detect specific vibration patterns in cells [19, 20], oocytes at different developmental stages [21], antibiotic‑resistant bacteria [22], brain tumor tissues [23], and epithelial‐mesenchymal transition in cells [24]. Collectively, these studies demonstrate that monitoring nanomechanical vibrations offers a novel approach to profile the dynamic biological state of living systems. However, current AFM‐based nanomechanical research remains largely confined to monolayer cell cultures or cellular aggregates. These simplified models offer limited biological complexity and fail to capture patient‐specific therapeutic responses, which are crucial for personalized medicine. This gap hinders the clinical translation of this powerful biophysical sensing technology. In contrast, as a patient‐derived gastric organoid with a specific three‐dimensional architecture, it is likely to exhibit more complex mechanical behaviors compared to single‐cell [25], and better reflect patient‐specific sensitivity to drug treatment.

Based on this, our study integrates patient‐derived organoid models with nanomechanical vibration sensing to construct a drug screening and evaluation platform. We established gastric epithelial organoid models and systematically detected and analyzed the nanomechanical vibration characteristics of organoids, clarifying the relationship between vibration amplitude and organoid growth. Through a series of pharmacological perturbation experiments targeting the cytoskeleton (actin filaments, microtubules), cell‐cell junctions (via calcium chelation), and cell metabolism, we delved into the biophysical origins of the organoids' nano‐vibration signals. Furthermore, by performing Fourier transform analysis on the raw vibration signals, we identified characteristic frequency peaks and deciphered the specific impact patterns of different drugs on the vibration spectrum. To enhance the accuracy of signal analysis and recognition, we introduced a deep learning framework based on a convolutional neural network (CNN). Through data‐driven feature engineering, we achieved high‐precision classification of drug response. Finally, we applied this platform to evaluate the efficacy of the first‐line clinical chemotherapeutic drug paclitaxel on patient‐derived gastric cancer organoids. We successfully detected vibrational alterations at ultralow drug concentrations (pM level) that preceded morphological changes (97% accuracy). The predictions from the organoid mechanical model also faithfully reflected the impact of anti‐cancer drugs on both the nanomechanical vibration amplitude and frequency spectrum of the organoids. These results collectively validate the significant advantage of this platform for the highly sensitive, efficient, and non‐destructive screening of anti‐cancer drugs.

In summary, this study integrates patient‐derived organoids, nanomechanical vibration detection platform, deep learning analysis and organoid mechanical model, establishing a complete analytical workflow that includes vibration detection, AI‐based feature extraction, and drug response analysis. This work not only extends nanomechanical vibration detection to more complex three‐dimensional organoid models but also provides a technical framework to advance organoid‐based drug screening from static endpoint assessment toward dynamic, biophysical profiling, thereby holding promise for personalized cancer therapy and new drug development.

## 2 Results

### 2.1 Organoid Preparation and Nanomechanical Vibration Detection

**FIGURE 1:**
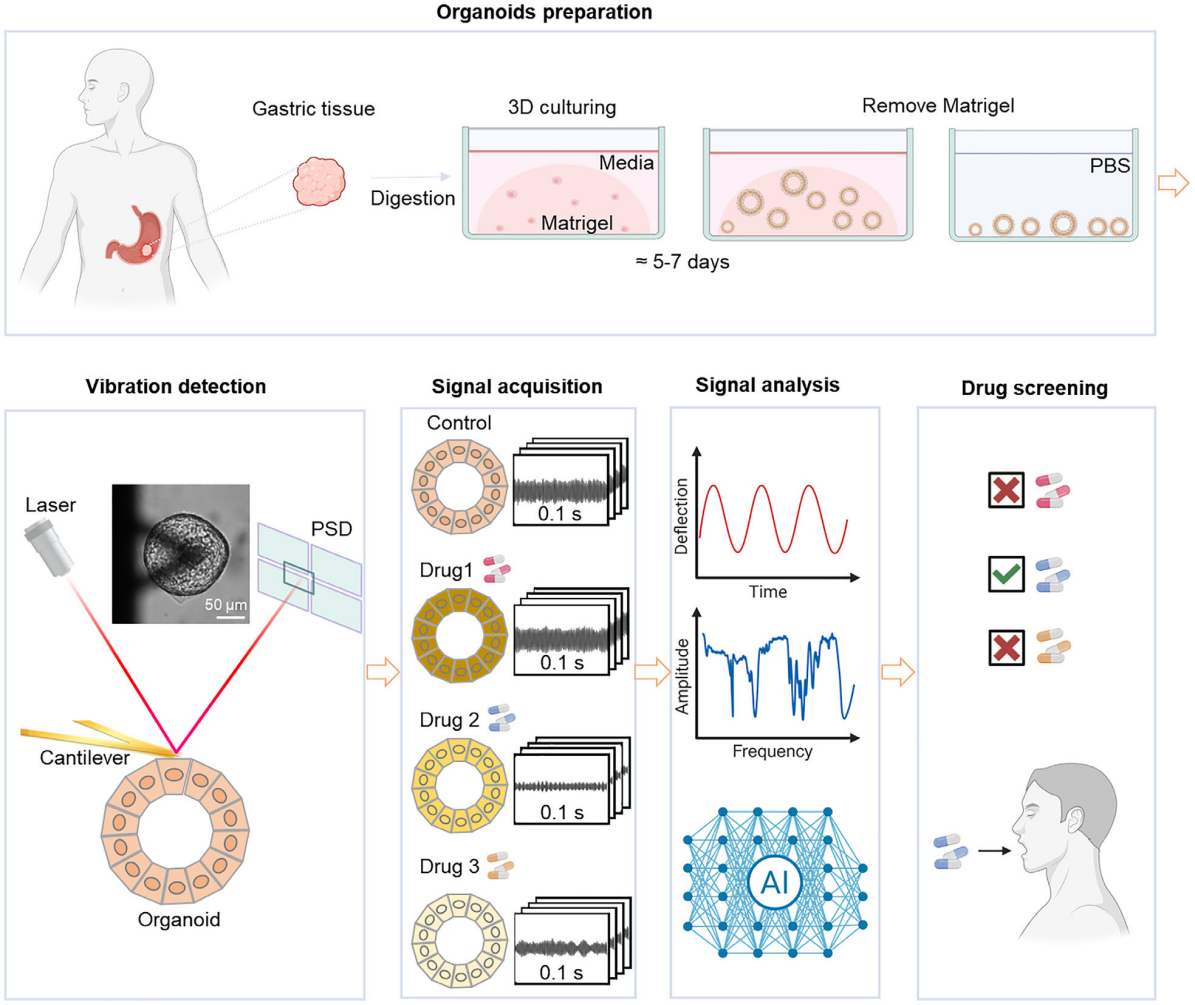
Schematic diagrams of organoid model construction and organoid nanomechanical vibration detection. Upper panel: Workflow for generating gastric organoids, from stomach dissection and enzymatic digestion of gastric glands to encapsulation in Matrigel for 3D culture. Mature organoids are then released by gentle pipetting. Lower panel: Nanomechanical vibration detection platform. Released organoids are transferred to a fibronectin‐coated dish in PBS. An atomic force microscope (AFM) NP‐O10 probe in Force Modulation Imaging mode contacts the organoid surface under a set force to record its vibration response. The platform analyzes both amplitude and frequency spectrum of the signals. Integrated deep learning‐based feature extraction correlates these vibration patterns with drug efficacy, highlighting its potential for personalized therapeutic selection.

The procedure for generating gastric organoids is illustrated in Figure 1 and Figure S1A. To establish organoids that recapitulate the structural and functional features of stomach, the gastric tissue was harvested from 8‐week‐old C57BL/6 mouse or gastric cancer patients. Gastric glands were isolated via enzymatic digestion and the resulting gland fragments were then embedded in Matrigel for three‐dimensional culture. Within 5 to 7 days, the gland self‐organized into hollow, spheroidal structures (Figure S1B). These mature organoids contained a distinct central lumen enclosed by a monolayer of highly polarized epithelial cells. The cells developed clearly defined apical and basal membrane, with the apical membrane contacting the lumen and the basal membrane interfacing with the surrounding Matrigel (Figure S1A). To further confirm the successful generation and multicellular composition of the organoids, immunofluorescence staining was performed using cell type‐specific markers: Lgr5 for stem cells, MUC5AC for surface mucous cells, and PGC for parietal cells. The positive staining for these markers verified that the gastric organoids comprised the afore‐mentioned multiple cell types, confirming the successful establishment of gastric organoids (Figure S1C).

To detect nanomechanical vibration signals, organoids were gently released from Matrigel by pipetting. The released organoids were washed and placed in 1×X  PBS, then transferred to the stage of an atomic force microscope (AFM; Bruker, JPK, Nanowizard). Measurements were performed using a tipless NP‐O10 probe in Force Modulation Imaging mode. The probe was brought into contact with the organoid surface until a preset indentation force (setpoint) was reached, then maintained at a constant position while its vibration signals were recorded, reflecting the subtle oscillatory activities within the organoids. Furthermore, we integrated deep learning analysis, which utilized features including vibration signal amplitude and frequency spectrum to detect and differentiate organoid states. Collectively, this nanomechanical vibration platform enables the assessment of drug responses in patient‐derived organoids, thereby holding promise for facilitating organoid‐based anti‐cancer drug screening and advancing personalized treatment strategies. The overall workflow is summarized in Figure 1.

### 2.2 Influence of Organoid Growth and Development on Organoid Nanomechanical Vibrations

**FIGURE 2:**
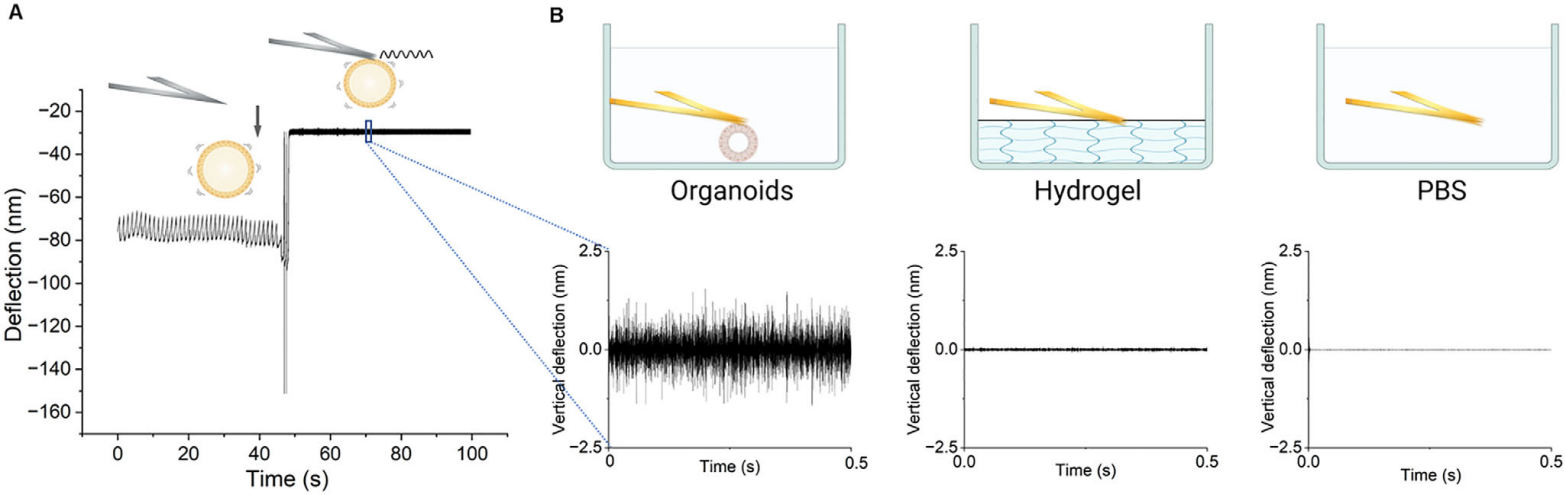
Nanomechanical vibration signals under different conditions. (A) Representative raw data of organoid nanomechanical vibration. The probe of the atomic force microscope gradually approached the organoid in automated mode at the beginning of the experiment. Contact with the organoid surface occurred at approximately 50 s, followed by the application of a predefined force. The vibration signal stabilized around 52 s and was continuously recorded. (B) Vibration signal recorded while probing a living organoid (Left panel). Signal recorded while probing a non‐living, 3 kPa polyacrylamide hydrogel (Middle panel). Signal recorded with the AFM probe hovering in PBS (Right panel).

As shown in Figure 2A, the original Time‐Deflection curve of the probe recorded the entire detection process. When the probe contacted the organoid and applied the preset indentation force (at approximately 50 s in Figure 2A), the AFM recorded a vibration. To confirm this vibration originated from the living organoid itself rather than environmental noise, we performed negative control experiments. Placing the AFM probe on the surface of a non‐living hydrogel or suspending it in PBS solution resulted in detected vibration amplitudes that were very weak (Figure 2B). In contrast, the nanomechanical vibration amplitude generated by the organoids was approximately 1.5 nm, significantly higher than that of the negative control groups (Hydrogel, 0.08 nm; PBS, 0.02 nm). This confirms that the vibration signal reliably reflects the intrinsic bio‐mechanical activity of the organoids.

**FIGURE 3:**
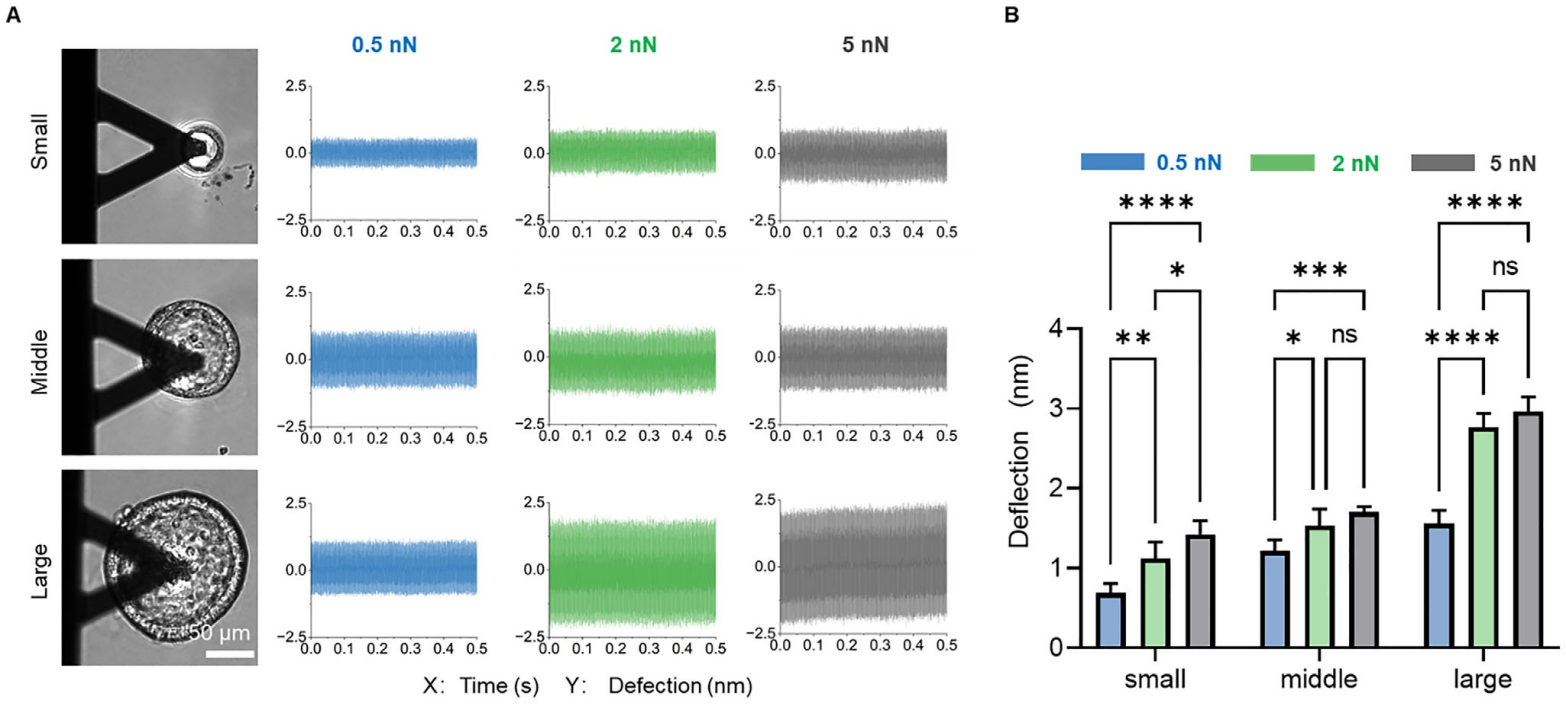
Organoid nanomechanical vibration amplitude correlates with size and preset indentation force. (A) Representative images depicting the nanomechanical vibration of organoids of different sizes. Organoids were categorized by diameter as small, middle, and large. The images show the vibration amplitudes of these organoids in response to different applied forces from the atomic force microscope probe (0.5, 2, and 5 nN). (B) Quantitative analysis of the data presented in Figure 3A. This graph summarizes the vibration amplitude of organoids, analyzing the effects of both organoid size and applied force. Data are presented as mean ± SD. Statistical significance was assessed using two‐way ANOVA. *n* = 4 for each group.

To investigate the changes in nanomechanical vibration signals during the growth and development of organoids, we conducted a systematic quantitative analysis (Figure 3). Organoids were categorized into three groups based on the different stage of growth: small (∼50 µm), middle (∼150 µm), and large (∼250 µm). Simultaneously, three levels of probe indentation force were set: 0.5, 2, and 5 nN. Vibration signals were detected under various combinations of size and indentation force, and the amplitude was quantified by the standard deviation of the vibration. The results revealed two key correlations: (1) under a constant indentation force, larger organoid size correlated with greater vibration amplitude; and (2) within the same size group, a higher indentation force led to an increase in the recorded amplitude. These correlations lead us to propose a plausible interpretation. The increase in vibration amplitude during growth may be attributable to cumulative effects from a greater number of actively vibrating cells, enhanced overall metabolic activity, and more efficient intercellular mechanical coupling, which could collectively enhance the mechanical signals transmitted to the probe. Regarding the force‐dependent response, we speculate that a higher indentation force likely reflects the global vibration of the entire organoid, whereas a lower force primarily captures minute vibrations generated by individual or small groups of cells at the surface.

In summary, these results demonstrate that non‐destructive and efficient nanomechanical vibration profiling can faithfully capture the authentic subtle vibrations of organoids, moreover, the nanomechanical vibrations of organoids change throughout organoid growth and this vibration‐based detection method is highly sensitive.

### 2.3 Pharmacological Perturbations Affect the Nanomechanical Vibrations of Organoids

**FIGURE 4:**
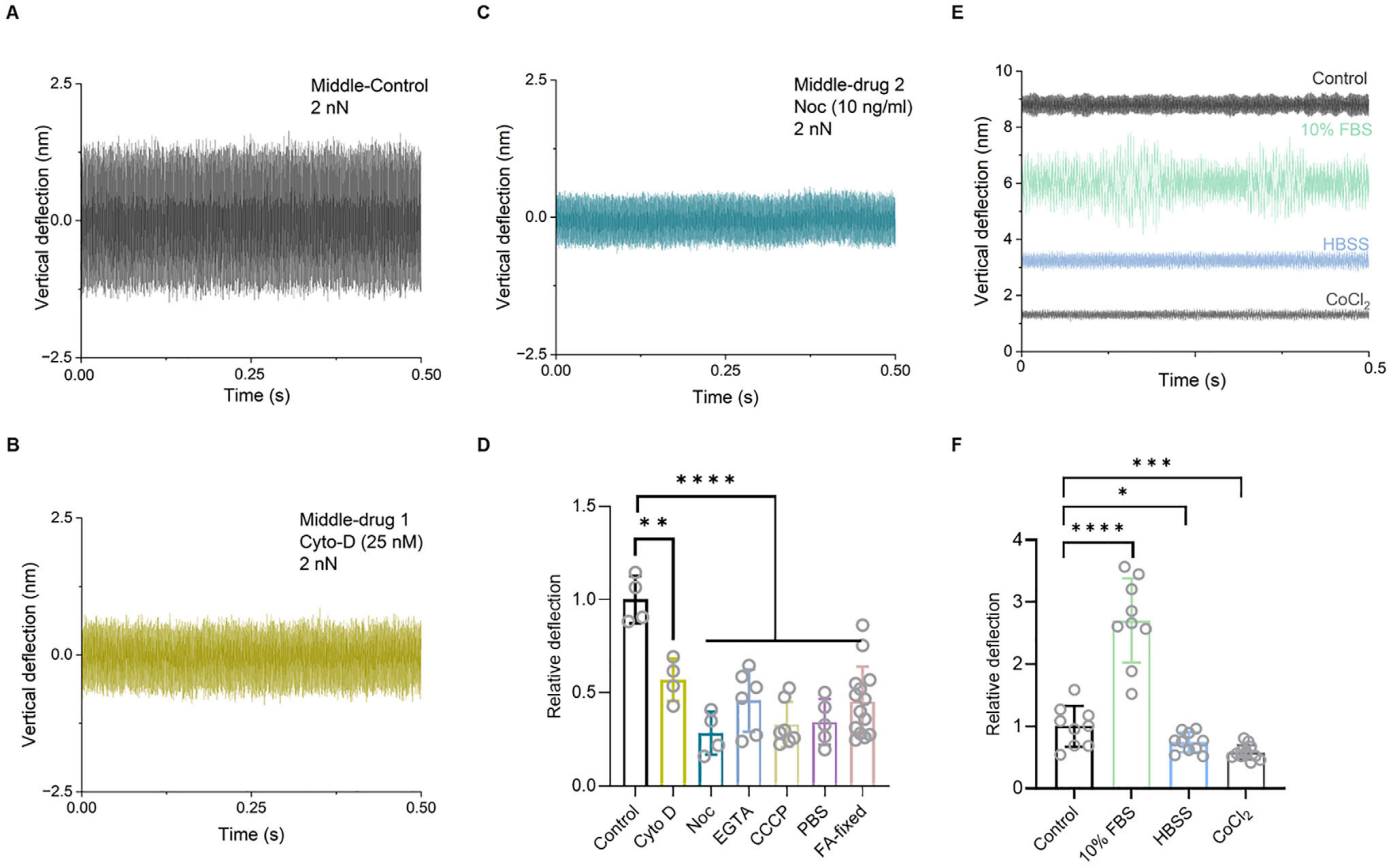
Pharmacological perturbations affect the nanomechanical vibration amplitude. (A–C) Changes in the nanomechanical vibration amplitude of gastric organoids under different drug interventions. Cyto‐D, Cytochalasin D, a drug that depolymerizes microfilaments, 25 nM; Noc, Nocodazole, a drug that depolymerizes microtubules, 10 ng mL^−1^. The organoids were pre‐treated with the drugs for 24 h, followed by nanomechanical vibration detection. (D) Statistical analysis of the data presented in Figure 4A–C and Figure S3 which reveal the impact of different drug on the nano‐vibration amplitude of organoids. PBS and formaldehyde‐fixed organoids (FA‐fixed) served as negative controls. Data are presented as mean ± SD; statistical significance was assessed using unpaired Student's t‐tests. *n* = 4 for Control, Cyto‐D, and Noc; *n* = 6 for EGTA; *n* = 7 for CCCP; *n* = 5 for PBS; *n* = 14 for FA‐fixed. (E) Representative images showing nanomechanical vibration amplitudes of gastric organoids treated with 10% FBS, HBSS, or CoCl_2_ (100 µM) for 24 h. (F) Statistical analysis of gastric organoids nanomechanical vibration amplitude after treatment with 10% FBS, HBSS, or CoCl_2_. Data are presented as mean ± SD (*n* = 9 for Control and 10% FBS group; *n* = 10 for HBSS group; *n* = 12 for CoCl_2_ group). Statistical analysis was performed using unpaired two‑tailed Student's t‑test.

To investigate the potential biological sources of the nanomechanical vibration signals in organoids, we conducted corresponding drug perturbation experiments. Subcellular structures within the organoids, including the cytoskeleton (actin filaments and microtubules) and intercellular junctions (e.g., E‐cadherin), serve as potential mechanical sources. Furthermore, mitochondria inside the cells, by mediating metabolism and sustaining cellular activity, may also influence the overall vibrational state (Figure S2A,B). To systematically explore the cellular origin of the vibration signals, we perturbed these substructures using specific pharmacological agents and detected the corresponding changes in the vibrational signals. The experimental results showed that treating organoids with low concentrations of an actin filament depolymerizer (Cytochalasin D, Cyto‐D, 25 nM) or a microtubule depolymerizer (Nocodazole, Noc, 10 ng mL^−1^) for 24 h significantly reorganized the cytoskeleton and mediated a reduction in their nano‐vibration amplitude (Figure 4A–C and Figure S2C).

Furthermore, other targeted drugs also produced effects (Figure 4D–F and Figure S3): treatments with EGTA (chelating calcium ions and interfering with cell junctions) [26], carbonyl cyanide m‑chlorophenyl hydrazone (CCCP, disrupting mitochondrial function) [27], HBSS (nutrient deprivation) [28, 29], and CoCl_2_ (simulating hypoxic environment) [30] all decreased the vibration amplitude of the organoids, whereas treatment with 10% FBS (promoting cell proliferation and metabolism) significantly increased the vibration amplitude.

The effects of these agents appear to be mediated by distinct mechanisms: Cyto‐D disrupts actin polymerization, dampening cytoskeleton‐driven motions; Noc depolymerizes microtubules, impairing long‐range mechanical signal transmission; EGTA chelates calcium to disrupt cell–cell junctions, reducing propagation of micro‐motions to the organoid surface; CCCP impairs mitochondrial ATP production, suppressing energy‐dependent vibrations; HBSS (nutrient deprivation) and CoCl_2_ (hypoxia) both limit energy supply, decreasing amplitude; whereas 10% FBS enhances metabolic activity, increasing amplitude. Collectively, these results demonstrate that organoid nanomechanical vibrations are highly sensitive to cytoskeletal integrity, intercellular coupling, mitochondrial function, nutrient and oxygen availability, and overall metabolic state.

### 2.4 Effects of Pharmacological Perturbations on the Vibration Spectrum of Organoids

**FIGURE 5:**
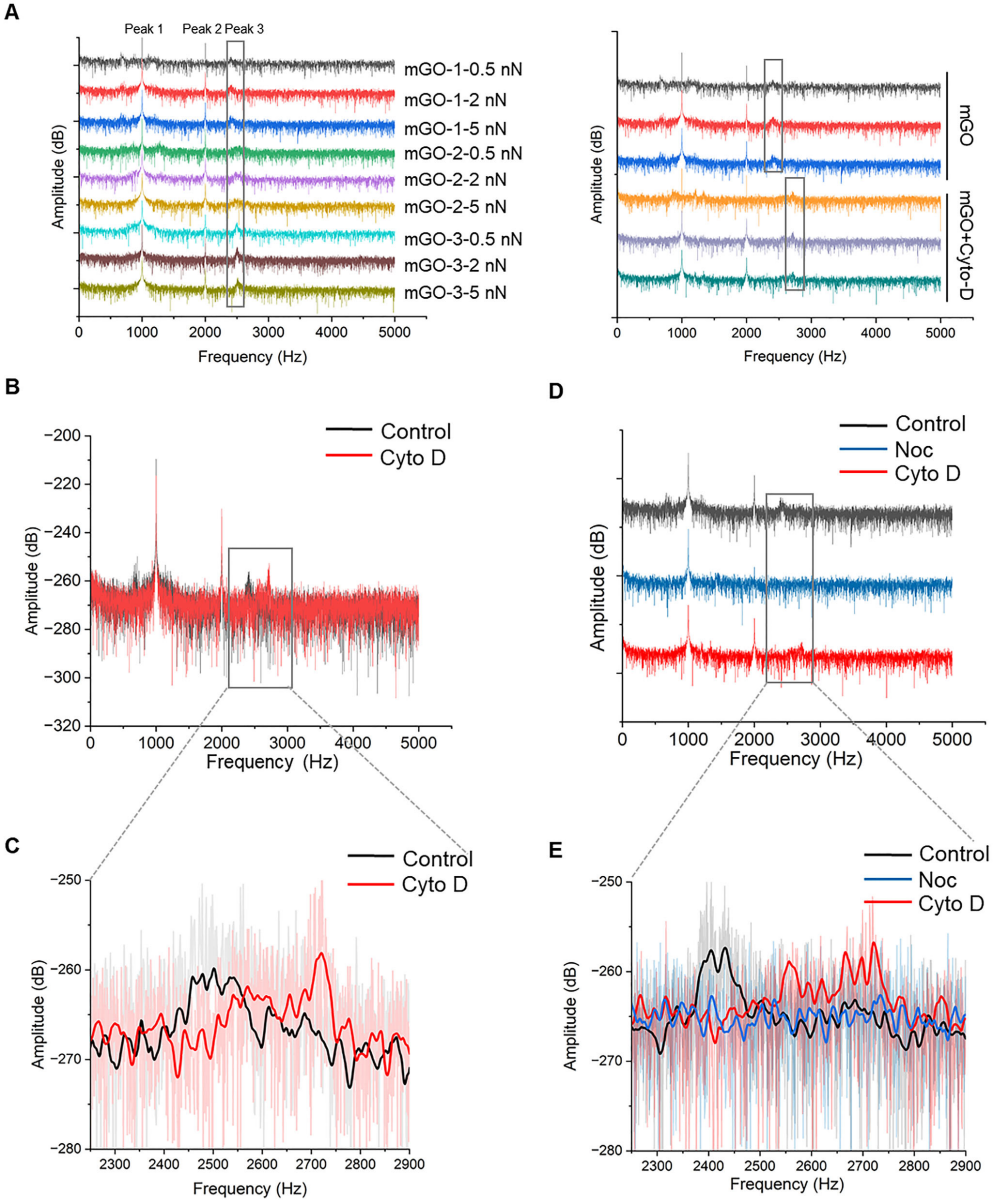
Organoid nanomechanical vibration spectrum. (A) Left: FFT‐derived spectrograms of organoid nanomechanical vibrations. A characteristic peak at ∼2500 Hz is highlighted (gray box). Spectra from three representative middle‐sized organoids under varying forces are shown (mGO, mouse gastric organoids), with replicates stacked (vertically offset) per group for clear visualization. Right: spectral changes of Peak3 in organoids following Cyto‐D treatment. (B) A stacked plot of the spectral data was generated to compare the organoid responses under Cyto‐D treatment, revealing positional change in the characteristic peak 3. (C) The magnified view of Figure 5B shows an alteration in the characteristic peak 3 following drug treatment. (D) Analysis of spectral data from organoids treated with Cyto‐D and Noc revealed position alterations in the characteristic peak at 2500 Hz, as highlighted by the gray box. (E) The magnified view of Figure 5D shows the positional change and disappearance of characteristic peak 3 following indicated drug treatment.

To investigate the impact of drugs on the mechanical properties of organoids, we performed Fast Fourier Transform analysis on the raw nano‐vibration signals from middle‐sized organoids. The spectral data revealed three stable characteristic peaks in the organoid vibrations: Peak 1 (980–1020 Hz), Peak 2 (1990–2010 Hz), and Peak 3 (∼2500 Hz). The presence of these peaks was independent of the applied AFM probe indentation force (Figure 5A). To rule out their origin as system artifacts, we performed control measurements by pressing the AFM probe against a non‐biological hydrogel. Spectral analysis of the hydrogel data revealed only Peak 1, with Peaks 2 and 3 absent. This contrast confirms that Peaks 2 and 3 specifically originate from the nanomechanical vibrations of the organoids (Figure S4A).

Given that nanomechanical vibrations reflect the minute movements of an organoid, and alterations in its internal structure are likely to modulate the vibration spectrum, we employed pharmacological perturbations to detect consequent changes in characteristic spectral peaks. Cytochalasin D (Cyto‐D) treatment resulted in the disappearance of Peak 3 and the emergence of a new peak to its right, while the Peak 2 remained stable (Figure 5A–C). In marked contrast, Nocodazole (Noc) treatment not only caused the complete disappearance of Peak 3 (Figure 5D,E) but also reduced the intensity of Peak 2—an effect not observed with Cyto‐D (Figure S4B,C). As the peak power reflects the vibration energy level driven by overall cellular activity [31], the specific attenuation of Peak 2 by Noc suggests its association with cellular metabolism and structural integrity. Overall, these results indicate that the two drugs act on distinct structural components within the organoids, and that the nanomechanical spectrum is sensitive to such microstructural alterations [32].

Collectively, these data indicate that pharmacological targeting of different cytoskeletal elements can alter organoid vibration patterns through specific mechanical pathways.

### 2.5 Deep Learning‐Based Ultra‐Sensitive Nanomechanical Vibration Analysis

**FIGURE 6:**
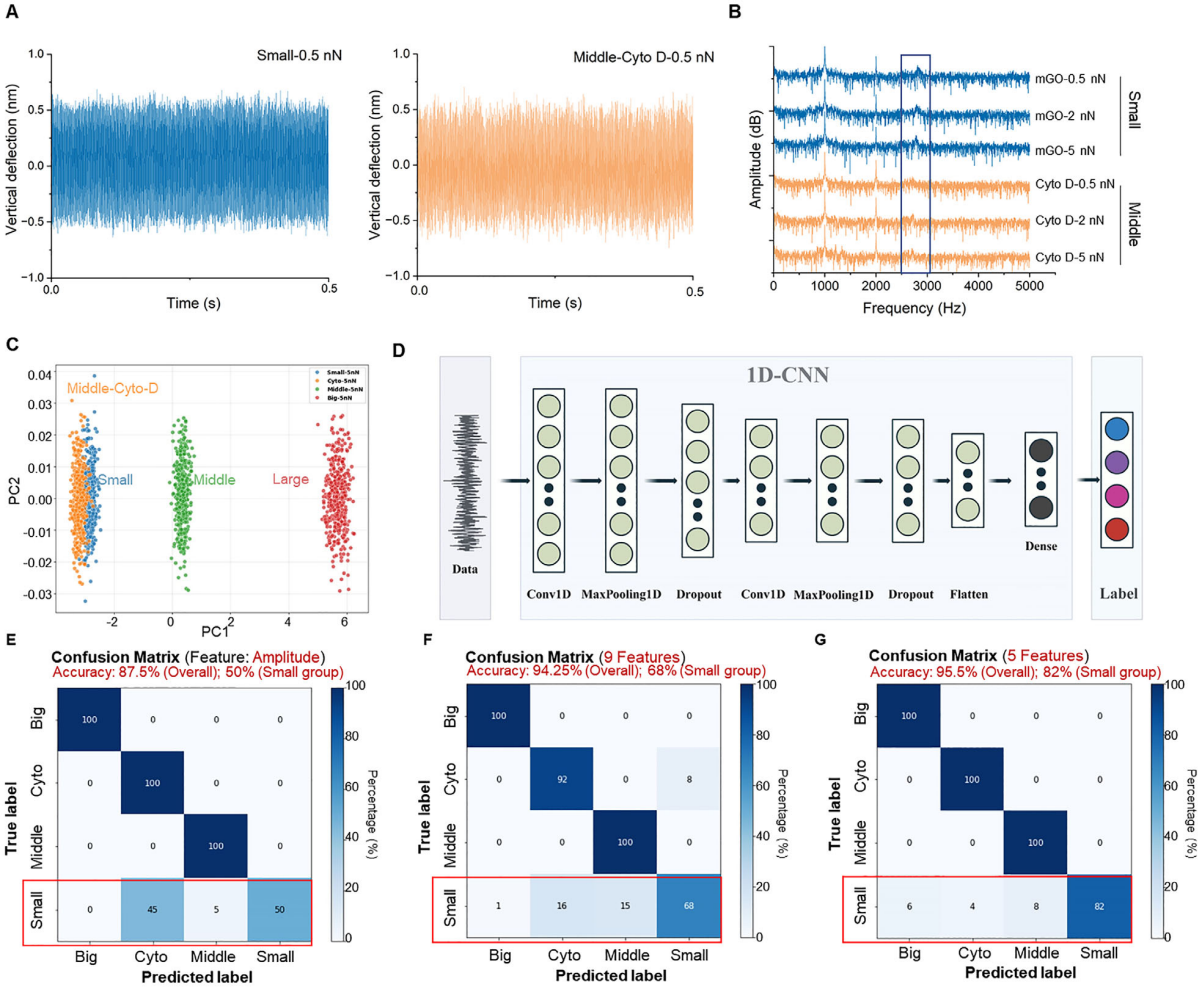
Overcoming organoid size heterogeneity via deep learning for precise classification of drug‐specific nanomechanical vibrations. (A) Nanomechanical vibration profiles of organoids from the Small (untreated) group and the Middle (Cyto‐D treated) group. (B) Nanomechanical vibration spectrograms from the untreated small group and the Cyto‐D treated Middle group of organoids. (C) Principal Component Analysis (PCA) was performed on the indicated four organoid groups: Middle‐Cyto D, Small, Middle, and Large. (D) Diagram of the one‐dimensional convolutional neural network (1D‐CNN) architecture. (E–G) Confusion matrix of the classification for 1D‐CNN using one feature (Amplitude) or nine features or optimized five features.

The nanomechanical vibration signals of organoids can be influenced by multiple factors, such as organoid size (Figure 3) and pharmacological perturbations, which alter both the amplitude (Figure 4) and frequency spectrum (Figure 5). As shown in Figure 6A,B, small organoids (Small group) and medium‐sized Cyto‐D treated organoids (Middle‐Cyto D group) were hardly to distinguish effectively based on amplitude or frequency spectrum shift. Principal Component Analysis (PCA) further confirmed that, when relying solely on amplitude features, these two sample groups exhibited significant overlap in the dimensionality‐reduced space (Figure 6C), indicating that a single feature is insufficient to fully characterize such a complex biophysical process. Therefore, highly sensitive analytical methods are required not only to detect these signal changes but also to disentangle the contributions of individual factors and even infer the underlying causes of specific alterations. Machine learning offers a powerful advantage in this context, demonstrating exceptional capability for efficient signal classification and for solving this type of inverse problem.

We first compared the classification performance of multiple machine learning models on the same dataset. The one‐dimensional convolutional neural network (1D‐CNN) demonstrated superior performance in both convergence speed and validation accuracy (Figure S5A,B). It also maintained the extremely high accuracy when analyzing groups that could easily be distinguished by amplitude and spectrum (Middle group and Middle‐Cyto D group) (Figure S5C,D), proving its advantage in capturing local and hierarchical patterns within vibration signals. Therefore, it was selected as the core model. Subsequently, we constructed a dedicated four‐layer 1D‐CNN network and applied it to classify the four groups: Big, Cyto (Middle+Cyto‐D), Middle, and Small (Figure 6D–G and Table S1). A key challenge was discriminating between the Cyto‐D and Small groups, as their amplitude and spectral profiles showed significant overlap (Figure 6A–C). When only the amplitude of nano‐vibration data was used as the model input, the overall classification accuracy reached to 87.5% (Figure 6E). However, the model struggled to differentiate the small group from the Cyto‐D group, achieving merely 50% accuracy, further highlighting the limitations of single‐dimensional amplitude information in capturing discriminative patterns of nano‐vibration signals. To address this issue, we leveraged domain knowledge of nano‐vibration physics to manually select nine vibration‐related features as inputs to the neural network model, which improved the overall validation accuracy to 94.25% and the accuracy for small group to 68% (Figure 6F). However, feature visualization analysis revealed that certain features (e.g., Peak Frequency) exhibited highly overlapping distributions across different categories (Figure S6), indicating weak discriminative power and the presence of feature redundancy or noise. Such redundant or non‐informative features not only failed to provide effective discriminatory signals but also increased input dimensionality, leading the model to suffer from “noise interference” during training: the neural network would erroneously attempt to fit these irrelevant features, diluting its focus on learning core discriminative patterns and ultimately compromising generalization ability or inducing overfitting. Further evidence from loss dynamics corroborated this issue: as shown in Figure S7, the validation loss of the model using the full nine‐feature set was notably higher than that of the model driven by the optimized five‐core‐feature set, a phenomenon that directly reflects the adverse impact of redundant features on model generalization.

To refine the feature set, we conducted data‐driven feature selection by calculating the absolute Pearson correlation coefficient between each feature and class labels, which quantifies the linear relationship between features and categorical outcomes. This process identified and eliminated redundant features, ultimately retaining five core discriminative features: Standard Deviation (STD), Mean Absolute Deviation (MAD), FFT Mean (FFT_Mean), Dominant Magnitude, and Skewness—all of which align with key physical attributes of nano‐vibration (e.g., STD reflects amplitude variability, FFT_Mean characterizes spectral energy) (Figure S6). Driving the convolutional neural network (CNN) model with this optimized, domain‐informed feature set significantly boosted the overall validation accuracy to 95.5% and the small group accuracy to 82% (Figure 6G). This improvement stems from the “purification” of input information: removing non‐discriminative features focused the model on meaningful physical patterns, eliminating ineffective learning on noise‐like features and enabling more efficient capture of the hierarchical and local patterns in nano‐vibration signals that distinguish different categories.

In summary, compared to traditional amplitude analysis and frequency spectrum analysis methods, ultra‐sensitive deep learning analysis can extract weak signals from complex signals in situations with large background noise, combined with feature optimization engineering, we successfully achieved high‐accuracy, size‐interference‐resistant classification of organoid nano‐vibration signals. The data‐driven feature selection improved model performance from 87.5% (Figure 6E) to 95.5% (Figure 6G), providing a sensitive analytical tool for organoids biophysics‐based drug screening.

### 2.6 Anti‐Cancer Drug Screening Utilizing Nanomechanical Vibrations of Organoids

**FIGURE 7:**
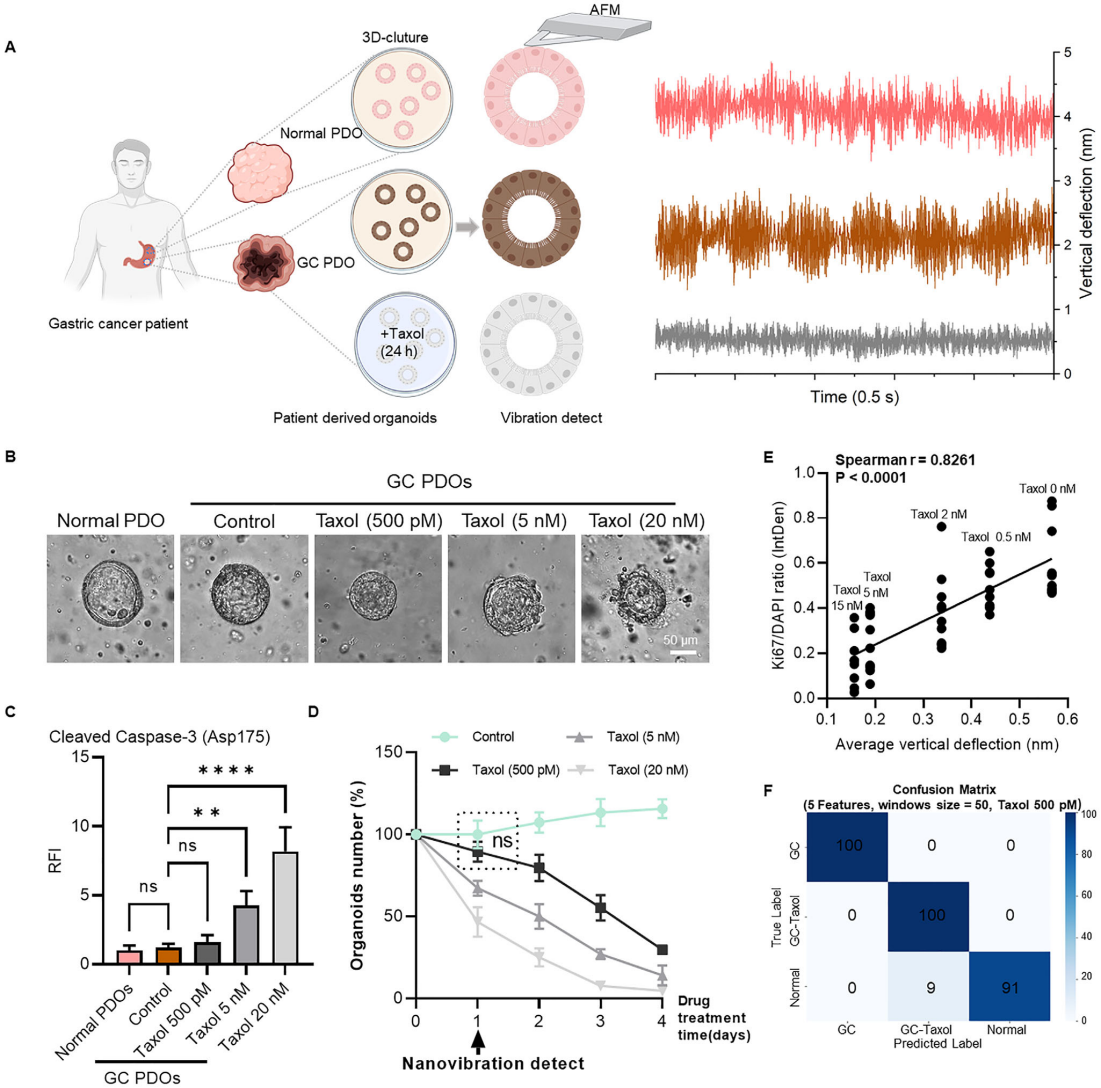
Nanomechanical vibration profiling of human normal and cancer gastric organoids. (A) Left, workflow for generating human normal and gastric cancer organoids (normal PDO and GC PDO) and nanomechanical vibrations detecting. Right, nanomechanical vibration profiling of normal PDO, GC PDO, and GC PDO with Taxol treatment. (B) Representative morphology of human normal PDOs, GC PDOs, and GC PDOs treated with paclitaxel for 24 h. Scale bar: 50 µm. (C) Statistical analysis from Figure S8B, showing apoptosis signals indicated by cleaved Caspase‐3 in organoids after drug treatment. Data are presented as mean ± SD; statistical significance was assessed using one‐way ANOVA. *n* = 4 for each group. (D) Extended culture of the different groups from Figure 7B, revealing viability outcomes following prolonged drug exposure. statistical significance was assessed using two‐way ANOVA. *n* = 4 for each group. (E) Spearman correlation between average vertical deflection and individual Ki67/DAPI ratio. Gastric cancer organoids were treated with five concentrations of paclitaxel (0, 0.5, 2, 5, and 15 nM) for 24 h. For each concentration, nanomechanical vibration were measured and the average vertical deflection was calculated. After vibration measurement, the same organoids were fixed and stained for Ki67, and the Ki67/DAPI integrated density ratio was quantified for each organoid. For correlation analysis, the mean vibration amplitude of a given concentration was paired with each individual Ki67/DAPI ratio from the same concentration (yielding 50 paired data points: 5 groups × 10 organoids), and Spearman rank correlation was performed. The analysis revealed a strong positive monotonic relationship (*ρ* = 0.8261, 95% CI: 0.7072–0.8996, *p* < 0.0001, *n* = 50). (F) Confusion matrix from the 1D‐CNN model classification of nanomechanical vibrations among human normal PDOs, GC PDOs, and GC PDOs treated with 500 pM paclitaxel. The model utilized the same five‐feature set as in Figure 6G with a window size of 50.

To advance the clinical translation of organoid‐based nanomechanical vibration detection for anti‐cancer drug screening, we further conducted drug testing on human gastric cancer organoids using established nano‐vibration detection platform. We isolated patient‐derived normal gastric mucosa and gastric cancer tissues from clinical surgical specimens. Through tissue digestion, filtration, and three‐dimensional Matrigel‐embedded culture, we successfully generated human gastric normal organoids (Normal PDO) and gastric cancer organoids (GC PDO) (Figure 7A). Morphologically, normal PDO exhibited the typical hollow, spheroidal structure with a clear lumen, whereas GC PDO showed disorganized architecture, and appeared irregularly cystic, which is consistent with previous reports [5, 33, 34] (Figure 7B and Figure S8A). We selected the first‐line clinical chemotherapeutic drug paclitaxel (Taxol) as the model anti‐cancer drug [35, 36]. Its mechanism involves promoting microtubule polymerization and inhibiting depolymerization, thereby arresting the tumor cell cycle and inducing apoptosis [37]. Morphological observation indicated that gastric cancer organoids maintained intact structure after 24 h treatment with 500 pM paclitaxel. However, when treated with 5 or 20 nM paclitaxel for 24 h, the organoids displayed obvious structural loosening and disintegration (Figure 7B). Staining with an anti‐cleaved caspase‐3 antibody also revealed that, unlike high‐concentration treatments, 500 pM paclitaxel treatment caused no significant increase in apoptosis signal. This aligns with the absence of morphological changes, indicating that low‐concentration treatment does not induce significant apoptosis or morphological disruption in the organoids [38, 39] (Figure 7C and Figure S8B).

To investigate whether nanomechanical vibration detection can sensitively capture the subtle effects of low‐concentration drug exposure, we performed nanomechanical vibration detection on normal PDOs, GC PDOs, and GC PDOs (treated with 500 pM paclitaxel for 24 h). The results showed that compared to normal PDOs, GC PDOs had a higher vibration amplitude (Figure 7A), which is consistent with marked cytoskeletal reorganization (increased microfilament and microtubule intensity) and enhanced glucose uptake (2‐NBDG), suggesting that the elevated amplitude arises from cytoskeletal reinforcement and hypermetabolic activity (Figure S8C,D). Notably, after paclitaxel treatment, the amplitude of gastric cancer organoids markedly decreased (Figure 7A and Figure S9). We extended this observation to gastric cancer organoids derived from another seven independent patients (Figure S10): paclitaxel (500 pM, 24 h) consistently reduced the vibration amplitude across all seven GC PDO lines, albeit with varying degrees (30%–70% reduction) reflecting interpatient heterogeneity. Furthermore, a concentration‐gradient experiment on Patient 7 (100 pM, 500 pM, 2 nM) revealed a clear dose‐dependent amplitude decrease. These results confirm that the paclitaxel‐induced amplitude reduction is reproducible across multiple patients, with the degree of reduction varying among individuals, further supporting the platform's utility in drug screening and its potential for precision medicine applications. Extended culture confirmed organoid death across all drug treatment groups (Figure 7D), this further demonstrates the high sensitivity of nanomechanical vibration detection, which can detect changes prior to any morphological alterations and cell death.

To substantiate vibrations as a reliable readout, we provide quantitative correlation data between vibration amplitude and classical cellular endpoint measured on the same set of organoids. Gastric cancer organoids were treated with five concentrations of paclitaxel (0, 0.5, 2, 5, and 15 nM) for 24 h. For each concentration, we measured the vibration amplitude of individual organoids (non‑destructive; Figure S11A, B), after which the same set of organoids were fixed and stained for the proliferation marker Ki67, and the Ki67/DAPI integrated density ratio was quantified (Figure S11C,D). Spearman correlation analysis revealed a strong negative monotonic relationship between paclitaxel concentration and each of the two parameters: vibration amplitude and Ki67 intensity (Figure S11E,F). Moreover, a strong positive monotonic relationship was found between vibration amplitude and individual Ki67/DAPI ratio (Figure 7E). These results directly demonstrate that the nanomechanical vibration signal quantitatively tracks proliferation status in a dose‑dependent manner, further supporting the central claim that nanomechanical vibrations serve as a reliable biological readout.

Relying solely on the amplitude of nanomechanical vibrations for drug efficacy assessment has limitations, including insufficient precision, lack of multidimensional insight, and inherent subjectivity. However, machine learning can analyze multiple data features beyond mere amplitude (Figure S6). Therefore, we applied our established CNN model, driven by an optimized five features (Figure S12), to classify the nanomechanical vibrations of organoids. The neural network successfully distinguished among normal PDOs, GC PDOs, and drug‐treated GC PDOs (Figure 7F), achieving an accuracy of 97%. Moreover, we have performed statistical power analysis on the human gastric PDO dataset, confirming that the sample size input into the model is sufficient to support the biological conclusions drawn in this study (Figure S13).

These results demonstrates that nanomechanical vibration profiling of organoids provides a more sensitive assessment of drug response than conventional endpoint assays. By integrating deep learning, this approach detects initial, subtle biophysical state changes that occur far earlier than macroscopic morphological alterations or cell death. As a sensitive, efficient, and non‐destructive process, it holds significant promise not only for the early and dynamic prediction of drug efficacy but also for enhancing preclinical screening to identify potent compounds, and ultimately for guiding personalized therapy (Figure S14).

### 2.7 Organoids Mechanical Model for Predicting Nano‐Vibration Changes

**FIGURE 8:**
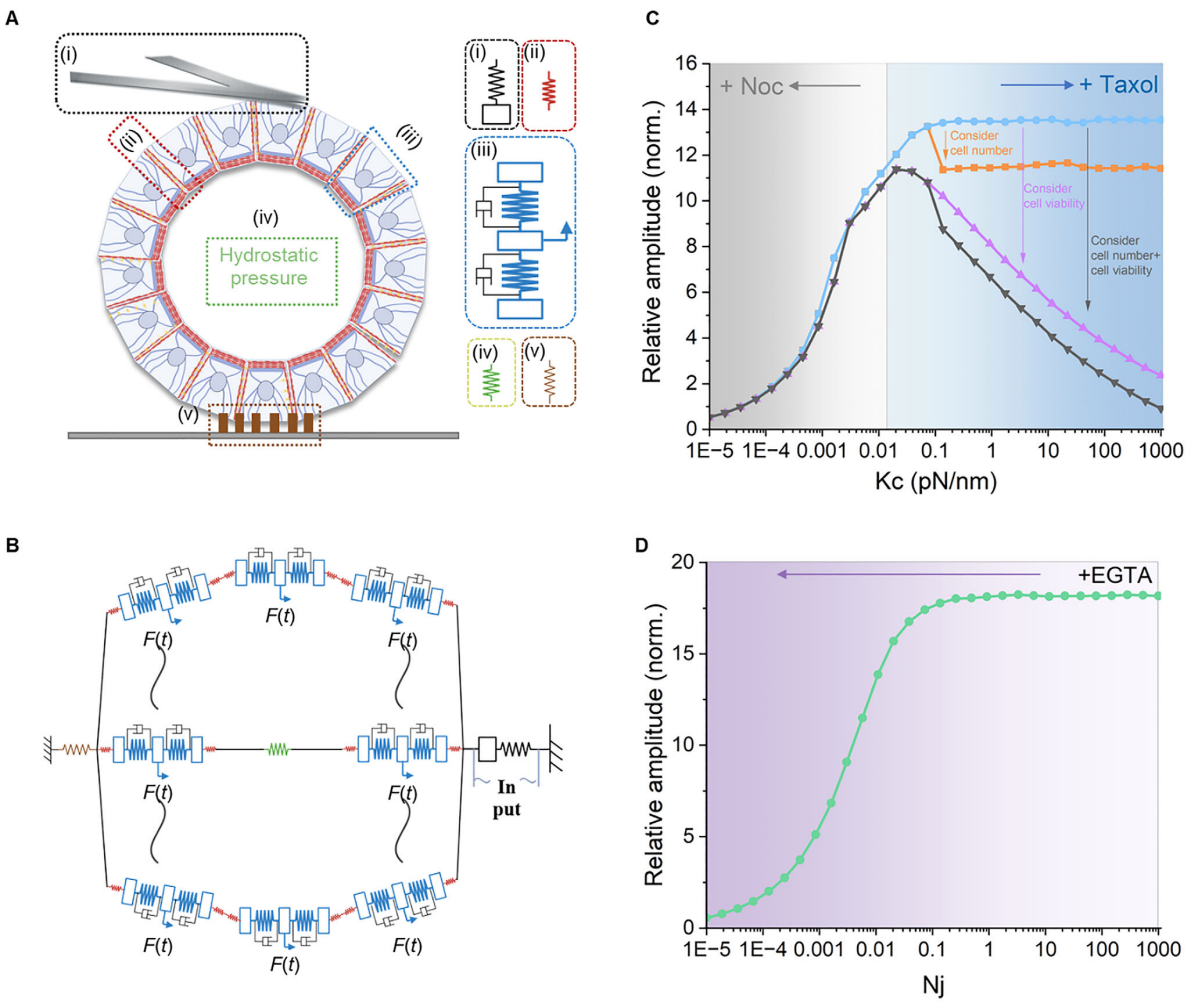
Organoids mechanics model and its prediction results for nano‐vibration. (A) Simplification of cell structures and microcantilevers in the organoid mechanical model: (i) microcantilever; (ii) cell−cell junctions; (iii) cell; (iv) hydrostatic pressure; (v) organoid‐matrix contact. (B) Organoid mechanics model considering cell vitality, cytoskeleton, cell‒cell junctions, hydrostatic pressure, and organoid‐matrix contact. (C) Using an organoid mechanical model to predict how alterations in microtubule dynamics induced by Paclitaxel/Nocodazole modulate cellular stiffness (Kc), thereby affecting the amplitude of nanomechanical vibrations in the organoids. The figure illustrates four distinct scenarios: drug treatment with no impact on cell viability (blue line), drug treatment affecting cell number (orange line), drug treatment influencing cell vitality (purple line) and drug treatment affecting cell vitality and cell number (black line). (D) Changes in the amplitude of nanomechanical vibrations in organoids were predicted by examining the effect of EGTA treatment, which reduces cell‐cell junctions (Nj).

To systematically predict the evolution of nanomechanical vibrations of organoids under pharmacological perturbations, we developed a mechanical simulation model. The key simplifications of the model are illustrated in Figure 8A,B: the microcantilever is treated as a mass‐spring element (Figure 8A, i); intercellular connections within the organoid are modeled as a series of springs (Figure 8A, ii); the organoid itself is represented as an actively driven mass‐spring‐damper system (Figure 8A, iii); intraluminal pressure‐mediated connections are also modeled as springs (Figure 8A, iv); and the adhesion of the organoid to the culture dish (anchored via fibronectin in experiments) is likewise modeled as springs (Figure 8A, v). All model parameters are detailed in Table S2.

Using this model, we successfully predicted the relative changes in nanomechanical vibration amplitude (Figure 8C,D) and spectrum (Figure S15) of organoids following treatment with Paclitaxel (Taxol), Nocodazole (Noc) and EGTA. Since both paclitaxel and nocodazole act on microtubules and consequently affect cellular cytoskeletal stiffness (the Kc value), we simulated their effects by modulating the Kc parameter in our model. The simulation revealed that enhancing cytoskeletal stiffness (Kc) increased the organoid vibration amplitude, while a sustained Kc increase eventually led to amplitude saturation, forming a plateau (Figure 8C, blue line). Given prior experimental evidence that drug treatment induces apoptosis in the gastric organoid epithelium, causing cell shedding into the lumen (Figure S8B), we incorporated drug‐induced reduction in cell number as a fixed parameter. This adjustment resulted in a decrease in the plateau intensity (Figure 8C, orange line). Furthermore, as high drug concentrations typically reduce cell viability, the model showed that incorporating drug‐induced loss of cell viability caused the amplitude plateau to disappear; instead, a continuous decline in amplitude was observed under sustained Kc increase (Figure 8C, purple line). Finally, by simultaneously considering the effects on both cell number and viability, the simulation predicted a more significant reduction in amplitude (Figure 8C, black line). These predictions align with our experimental results (Figure 4C and Figure 7A), confirming that under defined conditions, both paclitaxel and nocodazole reduce the nanomechanical vibration amplitude of organoids. We further simulated the effect of EGTA treatment, which disrupts intercellular junctions (Nj). By adjusting the Nj strength in the model, the prediction showed a declining trend in organoid amplitude with reduced Nj (Figure 8D), consistent with our earlier experimental data (Figure 4D). In summary, our constructed organoid mechanical model, which simplifies and simulates key mechanical components, effectively predicts the impact of pharmacological perturbations on organoid nano‐vibration amplitude. These perturbations alter the integrity of structural proteins, thereby modulating the transmission of mechanical signals within the organoid and consequently changing the vibration signals ultimately detected by the AFM probe.

Additionally, we simulated spectrograms of nanomechanical vibrations from the model and predicted the impact of drugs on organoid stiffness based on the spectrum shift: a leftward spectral shift indicates a weakening of surface stiffness, while a rightward shift indicates an increase. Based on this principle, the model successfully predicted changes in organoid stiffness under treatment with paclitaxel and EGTA (Figure S15). When simulating paclitaxel‐induced increase in Kc alone, the model predicted enhanced stiffness (Figure S15A). However, when incorporating the realistic effects of paclitaxel—reduced cell number and viability—the model predicted an overall decrease in surface stiffness (Figure S15B), further validating the model's accuracy in reflecting actual biological changes. Similarly, the model predicted a reduction in stiffness when simulating EGTA‐induced decrease in Nj value (Figure S15C). Overall, by linking predicted spectral shifts to potential mechanical changes, this model equips us with a capability to decipher how new drugs exert their therapeutic effects in future studies.

In summary, this mechanical model effectively predicted the regulatory roles of parameters such as the cytoskeleton and cell‐cell junctions on nanomechanical vibration amplitude and organoid stiffness, with the predicted trends being largely consistent with experimental observations. By achieving multi‐parameter coupling between cell viability and cellular structure, the model provides an analytical framework for explaining the generation mechanism of cellular nanomechanical vibrations from a biomechanical perspective.

## 3 Discussion

The emergence of organoid technology has provided unprecedented three‐dimensional in vitro models for disease modeling, developmental biology research, drug development and precision medicine [6, 40]. In this study, by integrating atomic force microscopy (AFM)‐based nanomechanical vibration detection, three‐dimensional organoid culture, deep learning analysis, and an organoid mechanical model, we constructed and validated an organoid nanomechanical vibration detection platform. Using this platform, we detected the nanomechanical vibration signals of organoids and monitored their dynamic changes throughout growth and development. Through pharmacological perturbation experiments, we identified the cytoskeleton and cellular metabolism as key biological sources of these signals. Furthermore, by employing deep learning‐driven feature optimization, we significantly improved the platform's analytical performance. Finally, we applied this optimized platform to patient‐derived gastric cancer organoids. It sensitively detected the effect of paclitaxel at an ultra‐low (pM) concentration, preceding any observable morphological changes or cell death. Using the established deep learning model, we achieved 97% accuracy in distinguishing anti‐cancer drug efficacy. Consistently, the organoid mechanical model accurately predicted the drug's impact on organoid nanomechanical vibrations. Our work expands the research paradigm for organoid‐based drug screening—shifting from static, traditional biochemical analysis to dynamic, sensitive, efficient, and non‐destructive biomechanical monitoring. This platform holds potential as a new tool for organoid‐based personalized therapy and drug screening.

This platform possesses distinct advantages over traditional patient‐derived organoid based drug screening methods. First, it achieves high sensitivity. Traditional efficacy assessments often require days or even weeks to observe significant organoid disintegration or molecular marker expression [41]. Our results show that after paclitaxel treatment at concentrations as low as the pM range, although morphological changes are not yet significant, the nanomechanical vibration amplitude exhibits a clear decrease (Figure 7A). Combined with deep learning‐based feature extraction and optimization, our platform provides a valuable “time window” for highly sensitive drug efficacy evaluation. This advantage stems from the exceptional sensitivity of AFM in detecting nanoscale displacements, combined with the ability of deep learning to deliver high‐precision classification and powerful feature extraction. Second, the method is inherently non‐invasive and highly efficient. Compared to traditional biochemical assays that require organoids pre‐treatment procedures [33], our method enables non‐destructive detection of organoids. This makes it possible to conduct dynamic and continuous monitoring of drug efficacy in organoids. Moreover, post‐treatment nanomechanical vibration detection (including probe approach, contact, and signal acquisition) for a single organoid can be completed within 30 s (Figure 2A), while the minimum effective vibration data required for deep‑learning analysis is as short as 0.1 s, significantly boosting the efficiency of drug response screening. Third, in contrast to resource‐intensive genomic or multi‐omics profiling of patient‐derived organoids, our platform offers a more cost‐effective alternative that requires minimal sample material. Fourth, our system demonstrates its mechanistic inference capability. The nanomechanical vibration spectrum of organoids contains rich information that is mechanistically linked to drug action. Different pharmacological perturbations induce distinct alterations in these spectral profiles. Consequently, analyzing the drug‐induced spectral changes by combining deep‐learning analysis provides a pathway to infer the underlying mechanisms of drug action. In summary, our platform integrated the key advantages of enhanced sensitivity, non‐invasiveness, efficiency, and cost‐effectiveness, collectively paving the way for the organoid‐based drug screening and clinical translation.

This work also represents conceptual and methodological progress compared with previous studies based on 2D cell lines. Conceptually, our study targets patient‐derived 3D organoids; unlike 2D cell lines, organoids preserve strong tumor heterogeneity and drug‐specific sensitivity. By using the mechanical properties (vibration signals) of patient‐derived tumor organoids for anti‐cancer drug screening, our approach offers significant advantages over traditional biochemical methods, including high sensitivity, high efficiency, and non‐destructive detection. Thus, we have combined the optimal model for evaluating anti‐cancer efficacy—organoids—with a highly advantageous mechanical detection method, thereby greatly amplifying the strengths of both. Methodologically, on the basis of detecting and analyzing organoid nanomechanical signals, we integrated a deep learning algorithm with powerful feature extraction capabilities and high‐precision classification. Beyond using amplitude as a single feature, we expanded the types of features for recognition, removed redundant and weakly discriminative features, and retained the five most discriminative features, achieving a substantial improvement in overall accuracy (97%). In addition, we established an organoid‐specific mechanical simulation model to predict drug‐induced changes in the amplitude and spectrum of organoid nanomechanical vibrations. The incorporation of deep learning and mechanical modeling further enhances our ability to understand, analyze, and interpret organoid vibration signals.

Although this study has achieved preliminary outcomes, as an emerging interdisciplinary technology, several aspects require further in‐depth exploration and optimization. First, the investigation of nano‐vibration spectral information remains in its early stages. We have identified two stable characteristic frequency peaks; however, their precise cell biological origins remain unclear, and pharmacological perturbations alone may suffer from off‐target or indirect effects. Future work needs to combine more refined cell biological interventions (e.g., gene editing) and higher‐resolution spatial mechanical mapping (e.g., Peak Force Tapping mode) to establish quantitative causal links between spectral features and subcellular structures. Second, although our current platform achieves a more efficient drug screening strategy compared to conventional endpoint assays, there is a need to further enhance its throughput and standardization for rapid adoption. Current AFM detection remains a single‐point, relatively low‐throughput operation, limiting its potential for large‐scale drug screening. Looking forward, technical advances including parallel cantilever arrays, micro‐fabricated MEMS resonators, and automated positioning systems could substantially increase measurement throughput while maintaining nanomechanical sensitivity. Third, the generalizability of the present model across different AFM systems and laboratories has not yet been systematically evaluated. Differences in instruments, sample preparation method, and experimental batches may affect model performance. Future translational studies will be needed to systematically test the accuracy of our method across different platforms and laboratory settings, and that the deep learning model may require moderate parameter adjustment or retraining to maintain optimal performance. Finally, we acknowledge that the current dataset, derived from nine patients, represents a valuable starting point but remains limited in scale. Future validation in larger, multi‐center patient cohorts will be essential to further strengthen the clinical applicability and generalizability of the proposed platform.

Based on the foundation established in this study, this platform possesses broad prospects for development and application. First, given that our platform detects drug‐induced changes in cytoskeletal organization and cellular metabolism, processes that are commonly affected by diverse classes of anti‐cancer agents, it is conceptually applicable not only to conventional chemotherapeutics but also to targeted therapies and immunotherapies. The most direct application is its extension to the screening of novel anti‐cancer drugs and combination therapies. In the future, the platform can be used to systematically test the effects of various chemotherapeutic agents, targeted drugs, immunomodulators, and their combinations on patient‐derived tumor organoids [3, 42, 43]. Second, it can promote the construction of an organoid biomechanics atlas database based on nanomechanical vibrations. We can perform systematic nano‐vibration detection on gastric organoids derived from different stages of the gastritis‐to‐cancer transformation process and different cancer subtypes, collecting the vibration data, and integrating with multi‐omics information such as genomics and transcriptomics to build a “mechano‐genomic” correlation database. This database can then be linked to potential therapeutics, ultimately enabling a direct path from detecting patient‐specific nanomechanical vibration signals to personalized drug selection, thereby advancing precision medicine. Finally, its application can be explored in other biomedical fields. Beyond anti‐cancer drug screening, this platform may also be suitable for studying various cellular processes such as senescence, apoptosis, differentiation, and metabolism, as well as developmental biology, neurodegenerative diseases, and the rhythmic contraction mechanics of cardiac organoids. As the technology matures, the application scope of nanomechanical vibration will continue to expand.

In summary, this study developed an integrated platform combining AFM, patient‐derived organoids, deep learning, and organoids mechanical model. This system enables sensitive, non‐invasive, and effective monitoring of the effects of anti‐cancer drugs, offering promising potential to advance preclinical drug screening and precision medicine.

## 4 Conclusions

In conclusion, this study establishes an integrated nanomechanical vibration detection platform that combines patient‐derived gastric cancer organoids, atomic force microscopy, deep learning analysis, and an organoid mechanical model for sensitive, efficient, and non‐destructive anti‐cancer drug screening. Systematic pharmacological perturbation experiments revealed that the amplitude and spectral characteristics of organoid nanomechanical vibrations are sensitive to cytoskeletal activity, cellular metabolism, and developmental stages. In drug screening applications, the platform successfully detected vibrational alterations in gastric cancer organoids upon treatment with paclitaxel at picomolar concentrations—changes that occurred prior to observable morphological changes, cell death, or proliferation decline, demonstrating superior sensitivity over conventional endpoint assays. Through deep learning‐driven feature optimization, the platform achieved 97% accuracy in distinguishing drug efficacy, while the organoid mechanical model accurately predicted drug‐induced changes in vibration amplitude and spectral features, providing mechanistic interpretability. Compared with existing methods, our platform offers distinct advantages including high sensitivity, non‐invasive and rapid detection, cost‐effectiveness, and mechanistic inference capability, shifting organoid‐based drug screening from static, destructive biochemical readouts toward dynamic biophysical profiling. We acknowledge several limitations that warrant future investigation, including the precise subcellular origins of specific spectral peaks, throughput constraints, drug diversity, and validation in larger patient cohorts—all of which represent our key directions for future work. Overall, this work establishes a new technical framework for biomechanics‐based organoid drug evaluation and screening, and holds promise for accelerating personalized cancer therapy and preclinical drug development.

## 5 Materials and Methods

### 5.1 Generation of Mouse Gastric Organoids

Mouse gastric organoids (mGO) were generated from gastric glands isolated from 8‐week‐old C57BL/6 mice. All procedures involving animals were approved by the Animal Ethics Committee of University of Science and Technology of China and adhered to the Guidelines for Animal Experiments of Laboratory Animals. The ethical approval number is 2025‐N(A)‐078. After euthanasia by CO_2_ asphyxiation, the stomach was excised, everted, and rinsed with ice‐cold Dulbecco's phosphate‐buffered saline (DPBS). The cardia and pylorus were ligated, and the stomach was distended with 500 µL DPBS, then incubated in 5 mM EDTA‐DPBS at 4°C for 1 h with gentle shaking. The tissue was then manually agitated in dissociation buffer (containing 5.6 mM Na_2_HPO_4_, 8.0 mM KH_2_PO_4_, 96.2 mM NaCl, 1.6 mM KCl, 43.4 mM sucrose, 54.9 mM sorbitol, and 0.5 mM DTT, pH 7.0) for 2 min. After dissociation, the supernatant was centrifuged (150 g, 5 min, 4°C), and the pellet was resuspended in Matrigel (Corning, 354277). The mixture was plated in a 4‐chamber confocal imaging dish (Cellvis). After gelation (37°C, 20 min), warm culture medium (Suzhou XianJue Biotechnology, CM01271A) was added, and cultures were maintained at 37°C under 5% CO2.

### 5.2 Generation of Human Gastric Organoids

Human gastric cancer tissue specimens were obtained from surgical resections of patients after written informed consent and under a protocol approved by the Ethics Committee of The First Affiliated Hospital of USTC (Anhui Provincial Cancer Hospital), with all procedures performed in accordance with the Declaration of Helsinki and relevant guidelines, and the approval number 2026‑LLYJ‑0009. The tissue samples were immediately placed in ice‐cold primary tissue storage solution and processed within 2 h of collection. The gastric tissue specimens were transferred to a sterile petri dish, washed with ice‐cold DPBS to remove mucus and debris, and the mucosal tissue was minced into small fragments using sterile instruments. The minced tissue was digested in 5 mL of tissue digestion solution at 37°C for 20 min with shaking (150–200 rpm), with digestion monitored every 5 min and terminated by 5% FBS addition. The cell suspension was filtered through a 100 µm cell strainer, centrifuged, and the pellet was treated with red blood cell lysis buffer, washed with DPBS, and finally resuspended in Matrigel for culture initiation with complete medium. Tissue storage solution, tissue digestion solution, red blood cell lysis buffer, and human normal gastric epithelial organoid medium (K2004‐HG) were sourced from BioGenous Biotechnology. Human gastric cancer organoid medium (K211M02) was obtained from D1 Medical Technology.

### 5.3 Organoids Passaging

The passaging ratio was determined based on the density of the organoids. First, the Matrigel was removed using organoid recovery solution, and the organoids were digested into single cells or small cell clusters using organoid digestion solution. Finally, the cells were mixed with fresh Matrigel and the mixture was divided at an appropriate ratio for passaging. All reagents related were sourced from BioGenous Biotechnology.

### 5.4 Immunofluorescence (IF)

Mature gastric organoids cultured in four‐compartment live‐cell imaging chambers (Cellvis) were fixed with 3.7% (w/v) paraformaldehyde (Thermo Fisher, 28908) in PBS at 37°C for 20 min. After permeabilization with 0.2% (v/v) Triton X‐100 in PBS for 10 min at room temperature, the organoids were blocked with 1% (w/v) bovine serum albumin (BSA; Sigma, A7906) in PBS for 1 h at room temperature. Fixed organoids were incubated with primary antibodies in a humidified chamber for 1 h at room temperature or overnight at 4°C, followed by incubation with secondary antibodies for 30 min at room temperature. Nuclei were stained with DAPI (Sigma) for 2 min. Organoids were maintained in PBS, and image acquisition was performed using a Leica confocal microscope (STELLARIS). The following antibodies were used for immunofluorescence (IF) analysis: anti‐E‐cadherin (Cell Signaling Technology, 3195T), anti‐α‐Tubulin (Proteintech, CL488‐80762) and anti‐Myosin IIA (Cell Signaling Technology, 3403T). F‐actin was stained using Alexa Fluor 633‐conjugated phalloidin (Solarbio). Mitochondria w ere stained using MitoTracker dye (Beyotime, C1032). All secondary antibodies were obtained from Cell Signaling Technology.

### 5.5 Drug Treatment

To investigate the nanomechanical vibration amplitude alteration of organoids in response to pharmacological perturbations, we treated organoids on day 3 of culture with pharmacological inhibitors for 24 h before AFM detection (CCCP 1 h). The inhibitors, all obtained from MedChemExpress (MCE), included Cytochalasin D (25 nM), Nocodazole (10 ng mL^−1^), CCCP (5 µM), EGTA (100 µM) and Paclitaxel. For anti‐cancer drug paclitaxel, different concentrations were used as the figure indicated for 24 h before AFM detection. Cobalt chloride solution (CoCl_2_) was sourced from Sigma (15862‐1ML‐F).

### 5.6 Nanomechanical Vibration Detection of Gastric Organoids

All measurements were performed using an atomic force microscope (AFM; Bruker JPK NanoWizard). First, 0.5 µg mL^−1^ fibronectin solution (Sigma) was coated onto confocal culture dishes overnight at 4°C. The dishes were then gently washed three times with PBS (1 mL each) to remove excess protein, and finally inverted to air‑dry under sterile conditions. Organoids were released from Matrigel by gentle pipetting of the Matrigel‑organoid mixture using a micropipette. The released organoids were immediately transferred to the fibronectin‑coated and dried culture dishes. The dishes were then placed in an incubator for 10 min to allow firm attachment of the organoids to the dish bottom. Subsequently, the supernatant containing residual Matrigel was carefully removed and replaced with fresh PBS to avoid interference with AFM detection. A tip‑free NP‑O10 A probe was used in force modulation mode. Before signal acquisition, the cantilever was brought into contact with the organoid surface at a preset setpoint force. After reaching the setpoint, the probe was held at a constant position, and the vertical deflection signal was recorded for at least 10 s to capture the intrinsic nanomechanical vibrations of the organoids. Throughout the measurement, the microscopic system and the environment were kept relatively quiet and stable to avoid introducing extraneous interference signals.

### 5.7 1D‐CNN Model

A four‐layer 1D Convolutional Neural Network (1D‐CNN) was constructed to process fixed‐length signal feature vectors. The architecture utilized Rectified Linear Unit (ReLU) activation functions in all convolutional layers to introduce nonlinearity. To mitigate overfitting, a Dropout layer (rate = 0.25) was incorporated after each convolutional layer, followed by an additional Dropout layer (rate = 0.3) before the final fully connected layer. The model was trained using the Adam optimizer with an initial learning rate of 0.001 and the categorical cross‐entropy loss function. A dynamic learning rate scheduler was employed: the learning rate was halved if the validation loss did not improve for 5 consecutive epochs, and training was stopped early if no improvement was observed for 15 epochs, with the best‐performing model weights being restored. All implementations and computations were carried out using TensorFlow 2.x (Keras 2.10.0) on a system equipped with an NVIDIA GeForce RTX 5070 GPU.

### 5.8 Neural Network

The source code is detailed on the website: https://gist.github.com/guanglixueshiyanshi‐sys/546351cf458894add5a1950426586167

### 5.9 Statistical Analysis

All experiments were repeated independently at least three times (biological replicates). Data are presented as mean ± standard deviation (SD). For each experiment, the sample size (*n*) represents the number of organoids or independent measurements, as detailed in each figure legend. The specific statistical tests used for comparisons are also indicated in the corresponding figure legends. All statistical analyses were performed using GraphPad Prism 8 (GraphPad Software, San Diego, CA, USA) or OriginPro 2023 (OriginLab, Northampton, MA, USA). Statistical significance was defined as a two‑sided *p* value < 0.05. In figures, significance levels are indicated as *p < 0.05, **p < 0.01, ***p < 0.001, and ****p < 0.0001; ns indicates not significant.

## Author Contributions

**T.Z**. constructed the organoid system, performed the nanomechanical vibration, analyzed the data, and wrote the manuscript. **Q.C**. participated in the nanomechanical vibration detection experiments, performed the neural network analysis and mechanical modeling, and analyzed the data. **Y.Z**., **H.Y**., and **L.Z**. provided the gastric cancer samples. **S.L**. (**Shihai Lan**) and **Y.L**. participated in building the organoid mechanical model. **S.L**. (**Shuaiyu Liu**) and **Y.X**. participated in the experimental design. **S.W**. designed the experimental protocol, and reviewed and edited the manuscript. **Q.Z**. conceived and designed the study, organized the research, and wrote the manuscript.

## Conflicts of Interest

The authors declare no conflicts of interest.

## Supporting information




**Supporting File**: advs76878‐sup‐0001‐SuppMat.pdf.

## Data Availability

The data that support the findings of this study are openly available in github at https://gist.github.com/guanglixueshiyanshi‐sys/546351cf458894add5a1950426586167.
