# Supplementary material for: Deciphering Anti‐Cancer Drug Efficacy Through Nanomechanical Vibrations in Living Gastric Cancer Organoids
Source: Adv Sci (Weinh). 2026 Aug 11:e76878. Online ahead of print. doi: 10.1002/advs.76878 (PMC13460407; doi:10.1002/advs.76878)
Supplement: Supplementary file 1 [file ADVS-9999-e76878-s001.pdf]

Deciphering Anti-cancer Drug Efficacy through Nanomechanical Vibrations in Living Gastric Cancer Organoids

Ting Zhang[a, #], Qiubo Chen[a, #], Shihai Lan[a], Liang Zhu[b], Shuaiyu Liu[c], Yan Xia[d], Yalu Zhang[b], Hanhui Yao[b], Yongman Liu[e], Shangquan Wu[a, *], Qingchuan Zhang[a, *]

[a] CAS Key Laboratory of Mechanical Behavior and Design of Material, Department of Modern Mechanics, University of Science and Technology of China, Hefei, 230027, China

[b] Department of Gastric Surgery, The First Affiliated Hospital of USTC, Division of Life Sciences and Medicine, University of Science and Technology of China, Hefei, 230027, China

[c] MOE Key Laboratory of Cellular Dynamics, CAS Center for Excellence in Molecular Cell Science, University of Science and Technology of China, Hefei, 230027, China

[d] Beijing University of Chinese Medicine Third Affiliated Hospital, Beijing, 100029, China

[e] School of Biomedical Engineering, Anhui Medical University, Hefei, 230032, China

[#] Ting Zhang, Qiubo Chen contributed equally to this work.

*Correspondence: wushq@ustc.edu.cn (Shangquan Wu); zhangqc@ustc.edu.cn (Qingchuan Zhang)

Figure S1

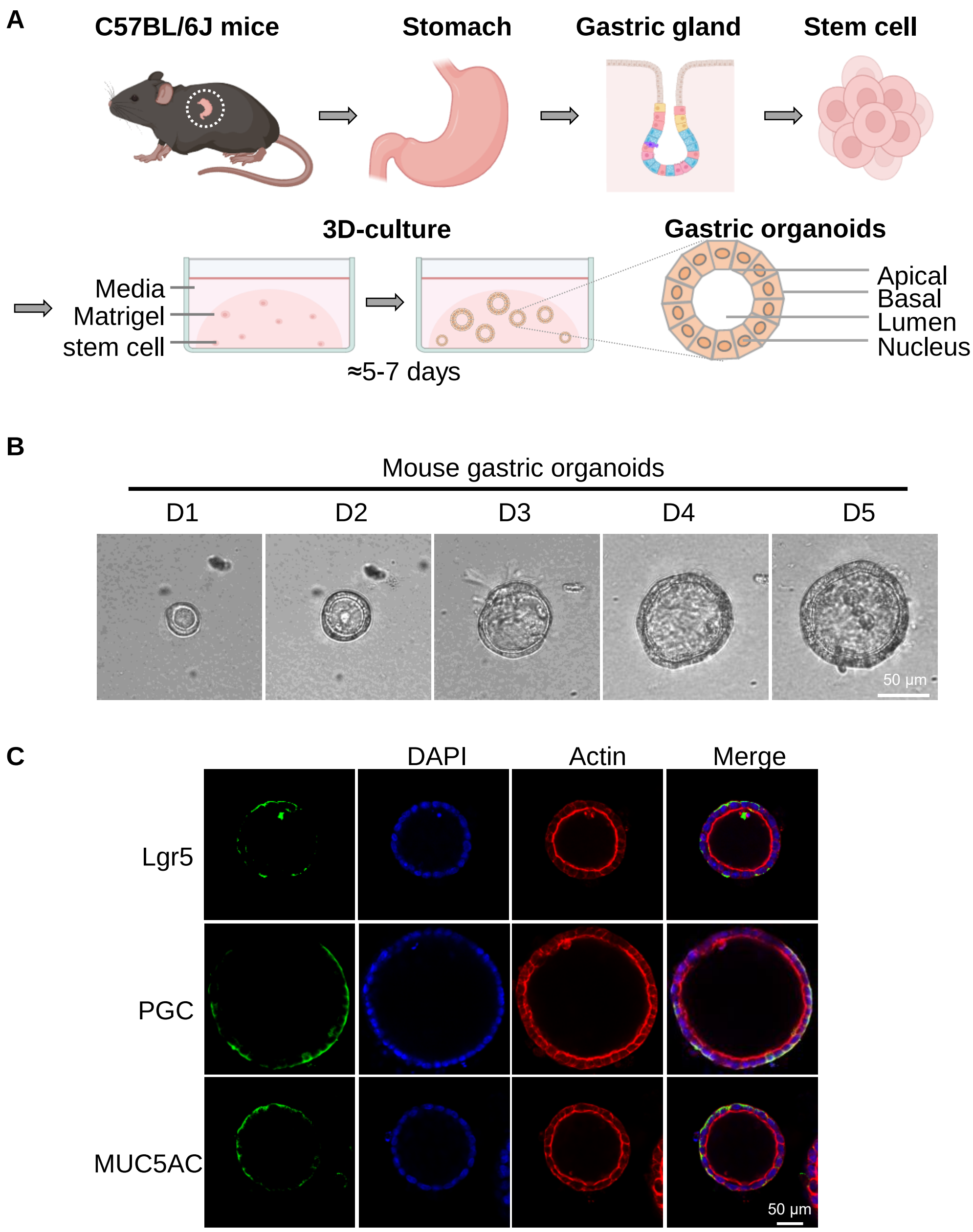

Figure S1 Representative images of organoid characterization and development.

A. Schematic diagram depicting the generation of mouse gastric organoids, involving stomach dissection, enzymatic digestion to isolate gastric glands, encapsulation in Matrigel, and subsequent 3D culture.

B. Time-course bright-field images showing the progressive growth and morphological maturation of the representative organoids over a 5-days culture period. Scale bar: 50 μm.

C. Mouse gastric organoids were immunostained with specific markers (Lgr5, MUC5AC, PGC) to confirm their multicellular composition. Scale bar: 50 μm.

Figure S2

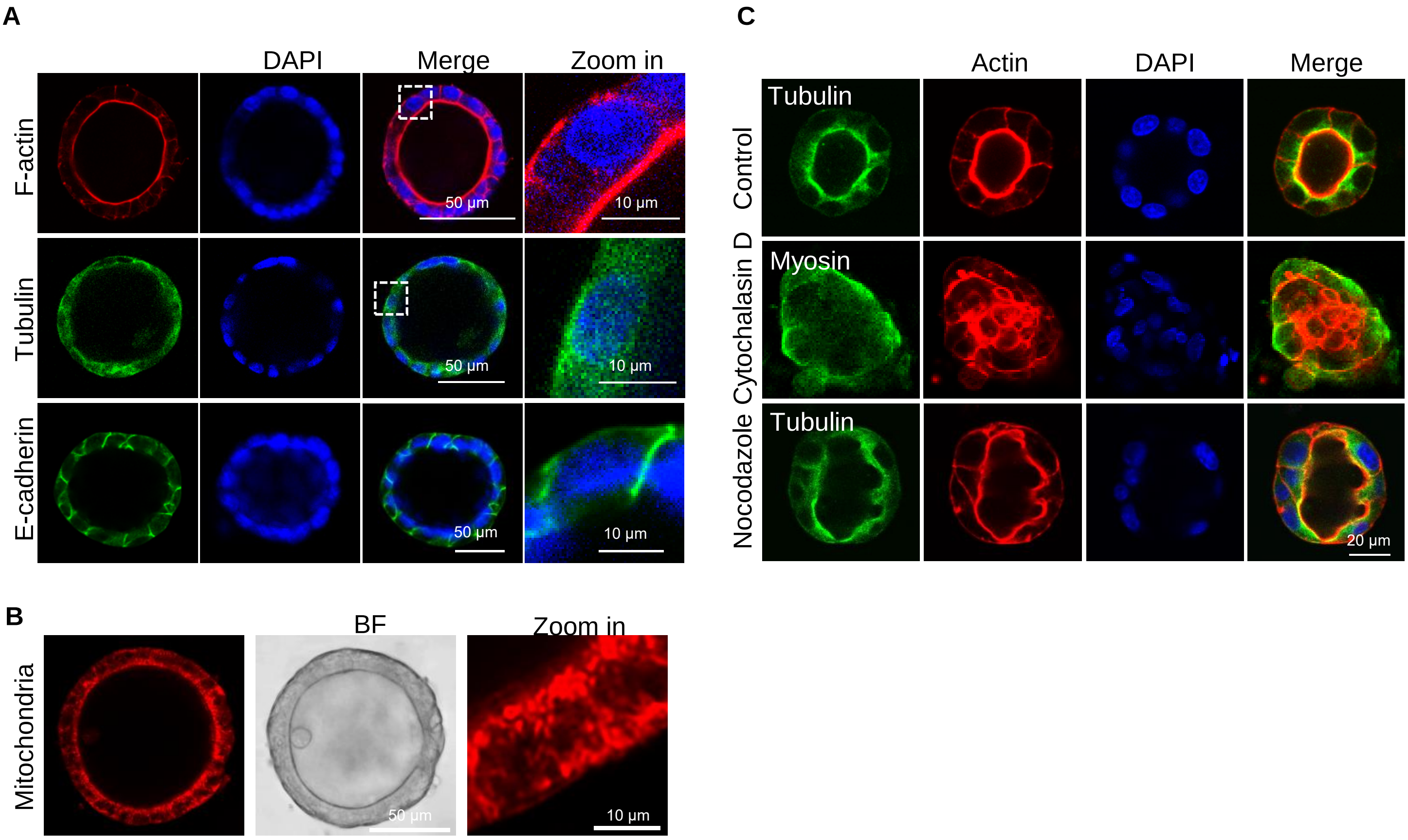

Figure S2 Representative images showing the distribution of key cellular structures in gastric organoids.

A-B. F-actin (phalloidin), microtubules (anti-α-tubulin), E-cadherin, and mitochondria (MitoTracker). Nuclei were counterstained with DAPI (blue).

C. Cytoskeletal distribution in organoids following pharmacological perturbation. Cyto-D, Cytochalasin D, a drug that depolymerizes microfilaments, 25 nM; Noc, Nocodazole, a drug that depolymerizes microtubules, 10 ng/mL.

Figure S3

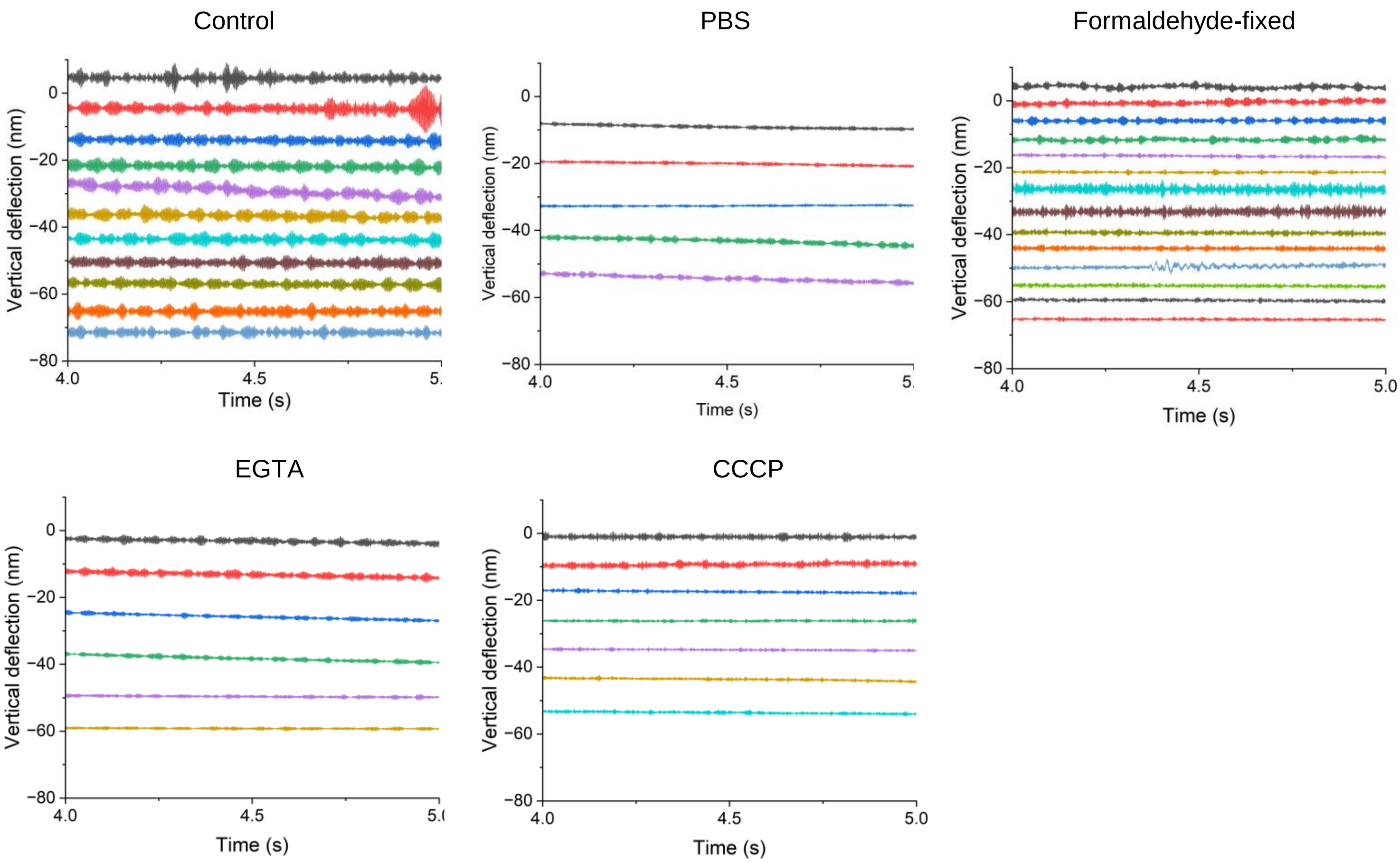

Figure S3 Nanomechanical vibration profiles of organoids under various pharmacological treatments. Representative 1-second waveform segments (from 10-second recordings) are shown for each condition, with individual biological replicates depicted in different colors. Treatments include: EGTA (100 µM, 24 h; calcium chelator), and CCCP (5 µM, 1 h; mitochondrial function inhibitor). PBS and formaldehyde-fixed organoids served as negative controls.

Figure S4

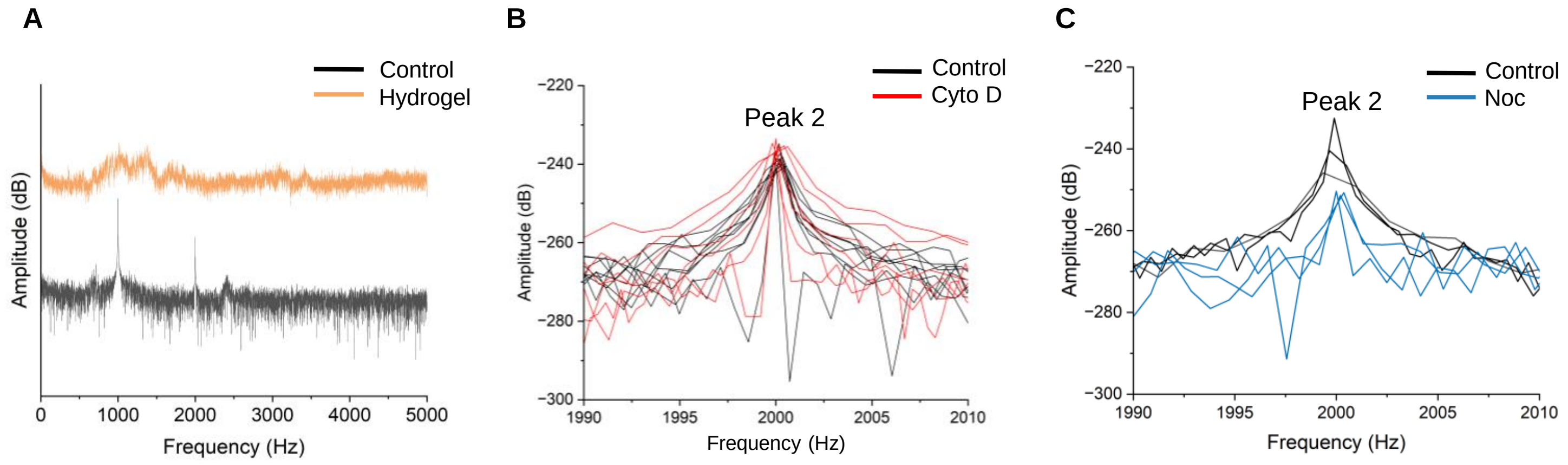

Figure S4 Organoid nanomechanical vibration spectrum under different drug treatment.

A. The acquired vibration detection signals from organoid (control) and gel (hydrogel) were subjected to Fourier transform for spectral analysis.

B. A magnified view of peak 2 highlights the difference in amplitude between normal organoids and those under Cyto-D treatment.

C. A magnified view of peak 2 highlights the difference in amplitude between normal organoids and those under Noc treatment.

Figure S5

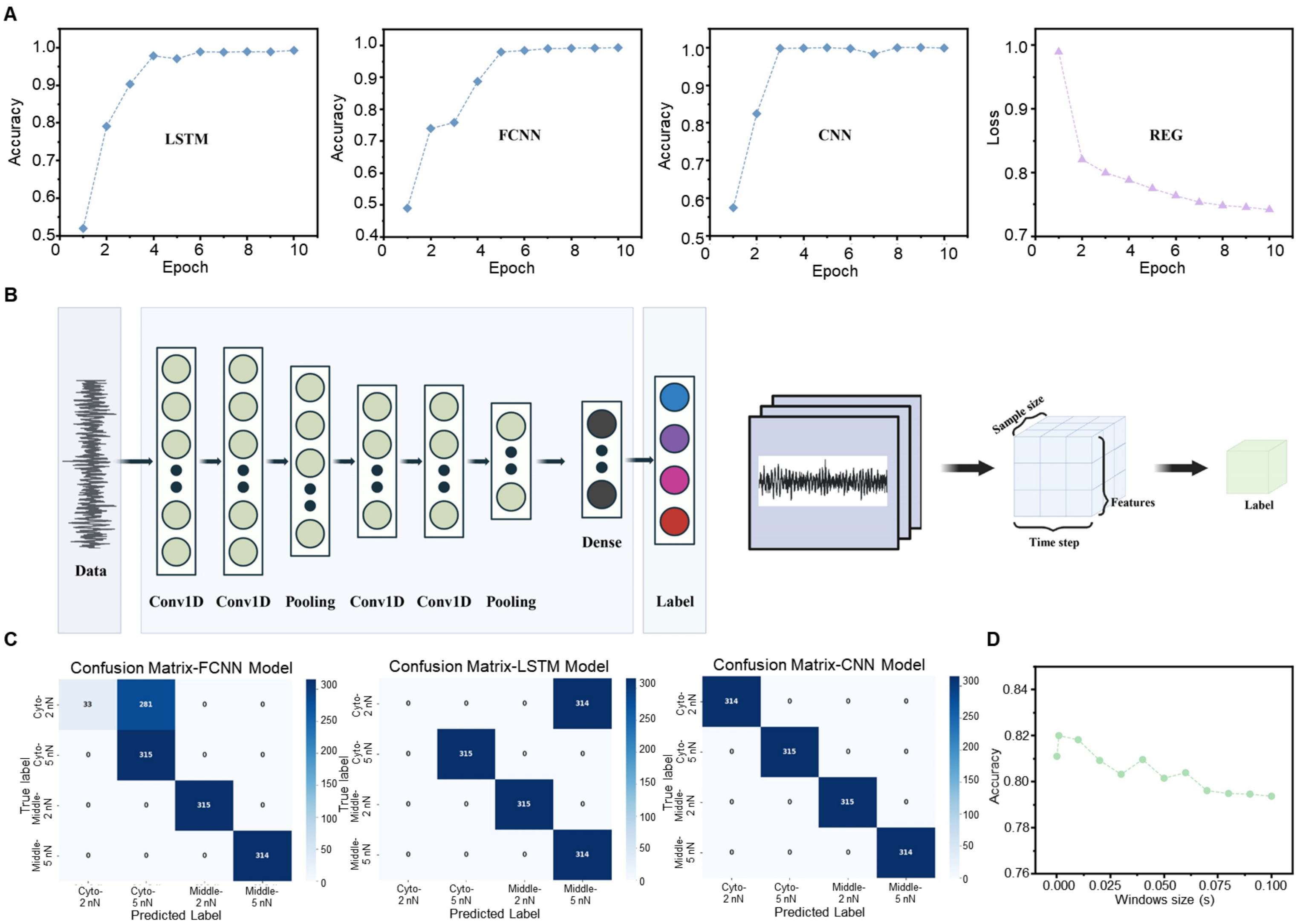

Figure S5 Classification performance of multiple machine learning models on the same dataset.

A. Classification accuracy of various machine learning models for nano-vibration signals.

B. Diagram of the machine learning models architecture.

C. Comparative performance of deep learning models in classifying Middle vs. Middle-Cyto D groups. The convolutional neural network (CNN) achieved the highest accuracy.

D. Effect of window step size on CNN classification accuracy.

Figure S6

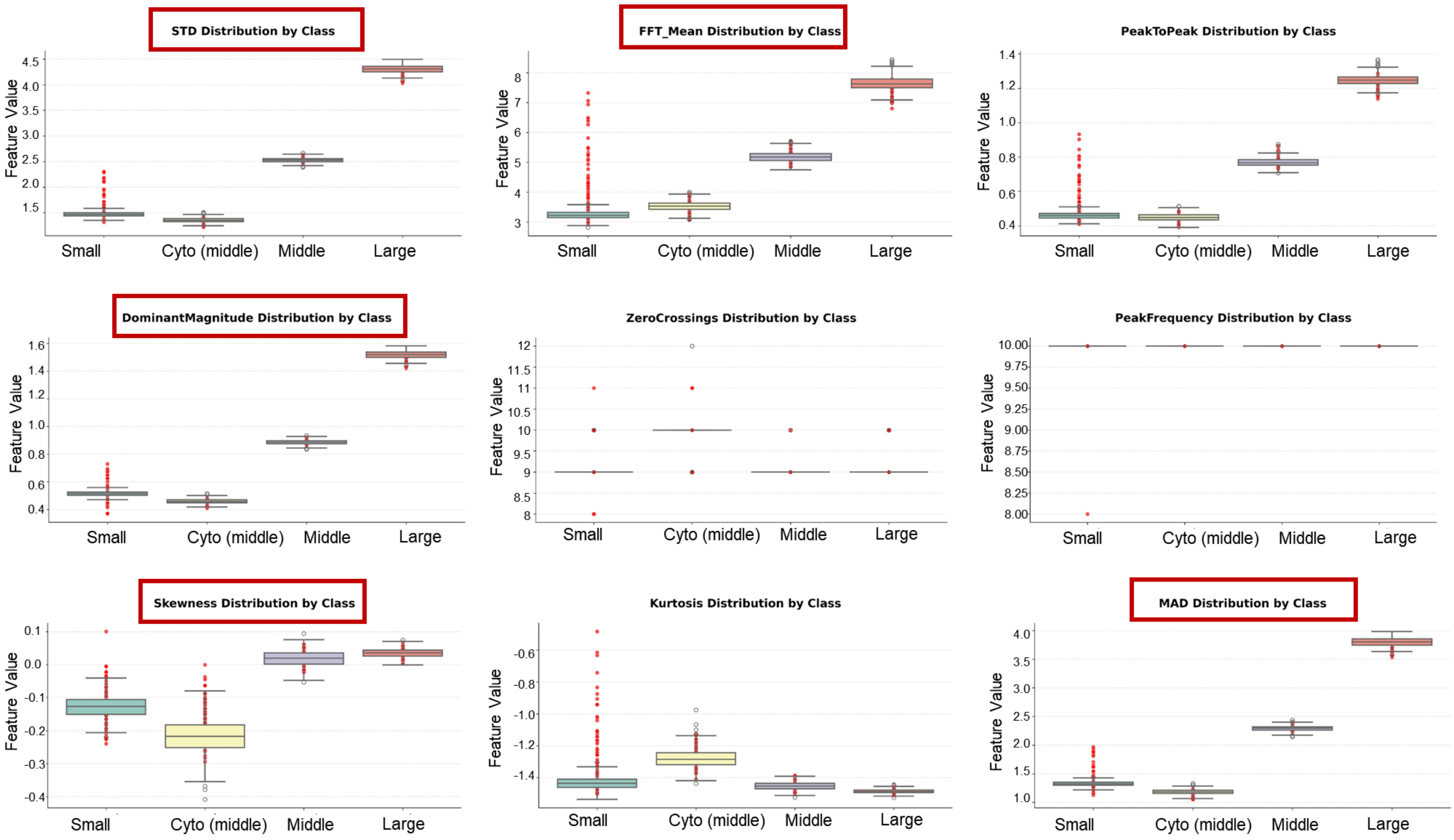

Figure S6 Nine features distribution across different organoid groups. Box plot showing the distribution of the nine features across the four organoid groups: Middle-Cyto D, Small, Middle, and Large. The box represents the interquartile range (IQR), the line inside the box denotes the median, and whiskers extend to show the variability outside the upper and lower quartiles. The features selected in the red box are optimized feature sets that differ significantly across these four sets of data.

Figure S7

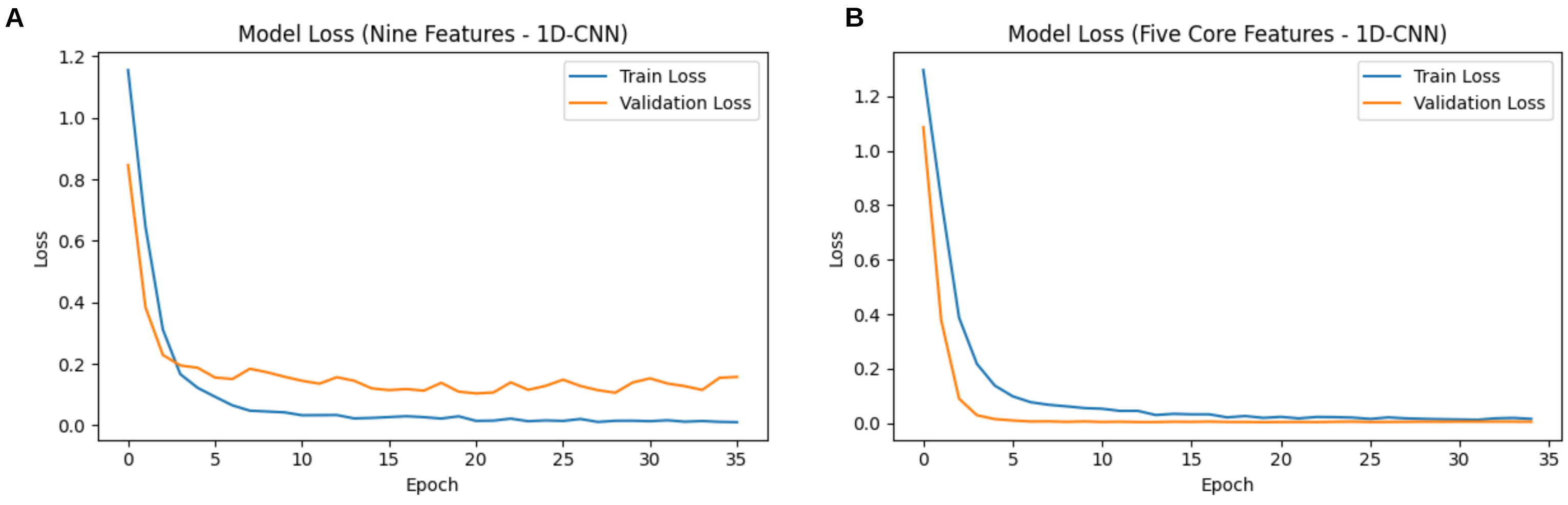

Figure S7 the validation loss of the model using the full nine-feature (A) or five-core-feature set (B).

Figure S8

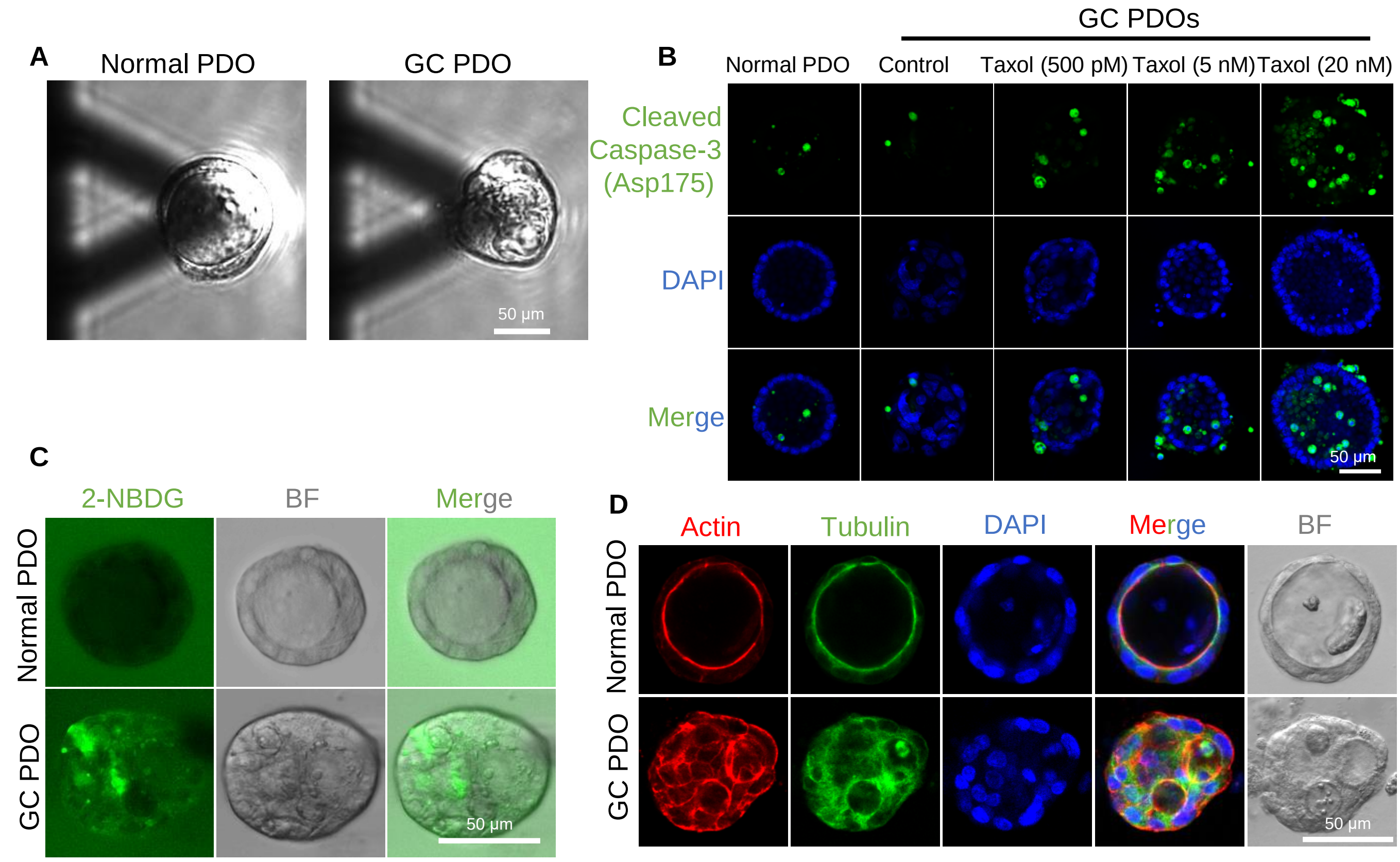

Figure S8 Characterization of morphology, cytoskeleton, glucose uptake, and drug-induced apoptosis in normal PDOs and GC PDOs.

A. Representative bright-field images showing the morphology of normal gastric organoids (left) and gastric cancer organoids (right) acquired during AFM measurements.

B. Dose-dependent induction of apoptosis in organoids by paclitaxel. Apoptosis was assessed via immunofluorescence staining of cleaved Caspase-3 (Asp175) after 24 hours of drug treatment.

C. Representative confocal images of normal PDOs (top) and GC PDOs (bottom) after incubation with 50 μM 2-NBDG (37 °C, 60 min). After thorough washing with PBS, 3D imaging was performed at 488 nm.

D. Fluorescence staining of F-actin (phalloidin, green) and α-tubulin (red) in normal gastric organoids (top) and gastric cancer organoids (bottom). Nuclei were counterstained with DAPI (blue).

Figure S9

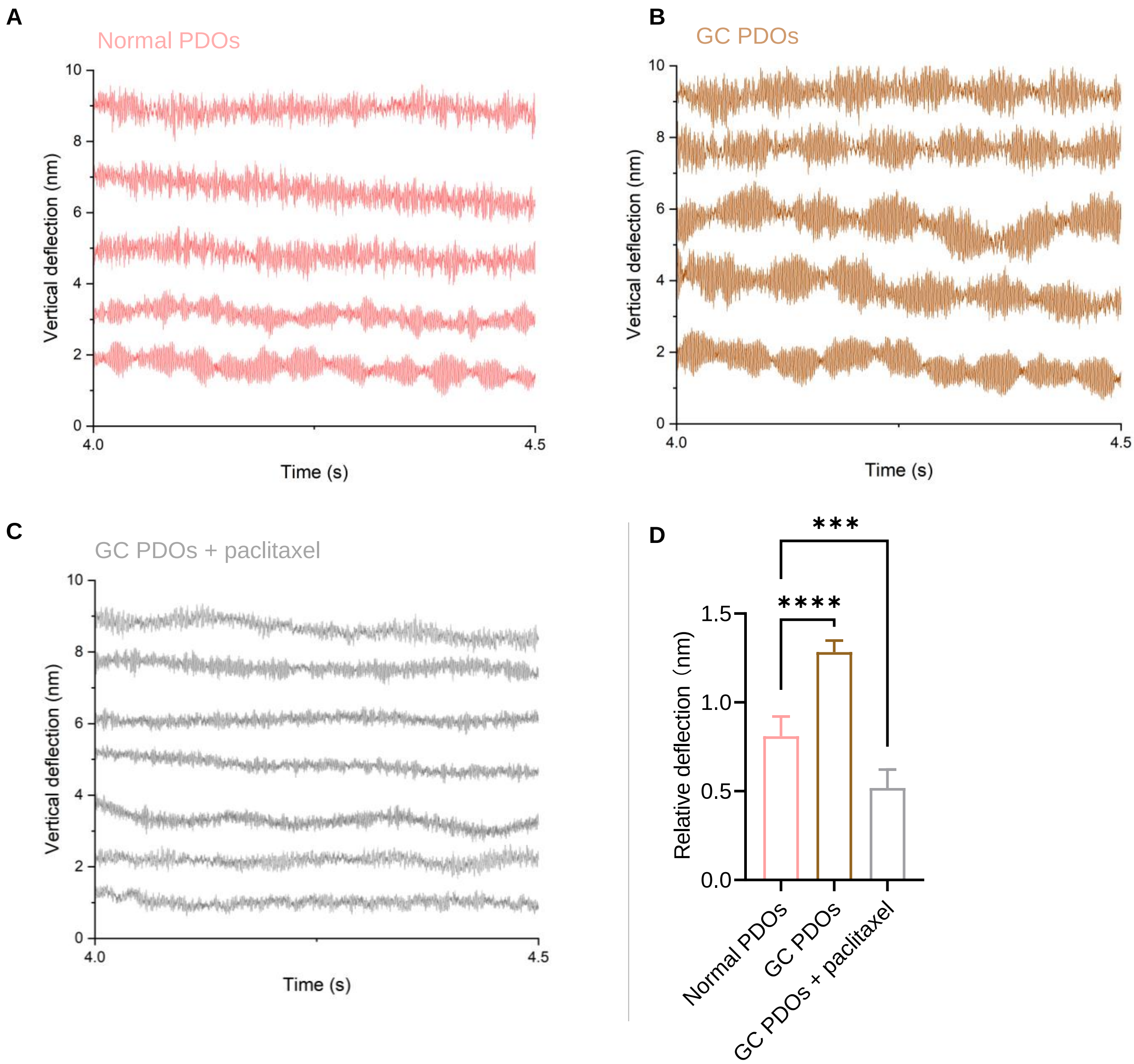

Figure S9 Effects of paclitaxel on nanomechanical vibration amplitude in organoids.
A. Representative raw nanomechanical vibration data from human normal PDOs, GC PDOs, and GC PDOs treated with 500 pM paclitaxel.
B. Statistical analysis of the data presented in Figure S9A-C. Data are presented as mean ± SD; statistical significance was assessed using unpaired Student‘s t-tests. Sample size: n = 5 for Normal PDOs and GC PDOs groups; n = 7 for GC PDOs + paclitaxel group;

Figure S10

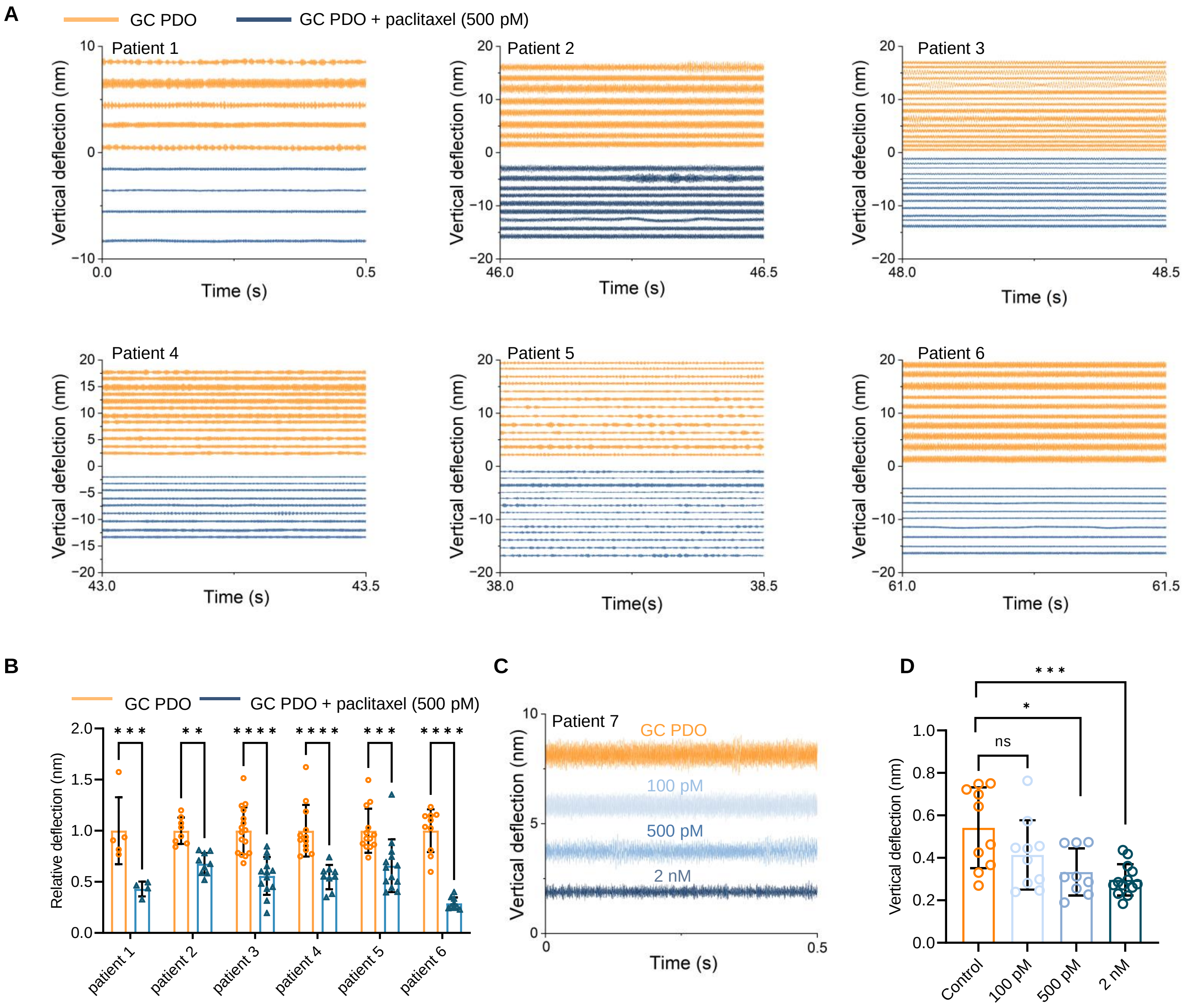

Figure S10 Effects of paclitaxel on nanomechanical vibration amplitude in organoids.

A. Raw nanomechanical vibration traces of human patient-derived gastric cancer organoids (GC PDOs) under control conditions and after treatment with 500 pM paclitaxel for 24 h (Patients1–6).

B. Statistical analysis of the data shown in Figure S10A. Data are presented as mean ± SD. Statistical significance was assessed using two-way ANOVA. For the control (GC PDO) group, the sample sizes (n) for Patients 1–6 were 5, 8, 16, 12, 13, and 10, respectively. For the paclitaxel-treated (GC PDO + paclitaxel) group, the sample sizes for Patients 1–6 were 4, 9, 13, 9, 13, and 9, respectively.

C. Representative raw nanomechanical vibration traces of GC PDOs from Patient 7 treated with the indicated concentrations of paclitaxel (100 pM, 500 pM, 2 nM) for 24 h.

D. Statistical analysis of the data shown in Figure S10C. Data are presented as mean ± SD. Statistical significance was assessed using unpaired Student‘s t-tests. Sample sizes: n = 10 for the control and 100 pM paclitaxel groups; n = 9 for the 500 pM paclitaxel group; n = 13 for the 2 nM paclitaxel group.

Figure S11

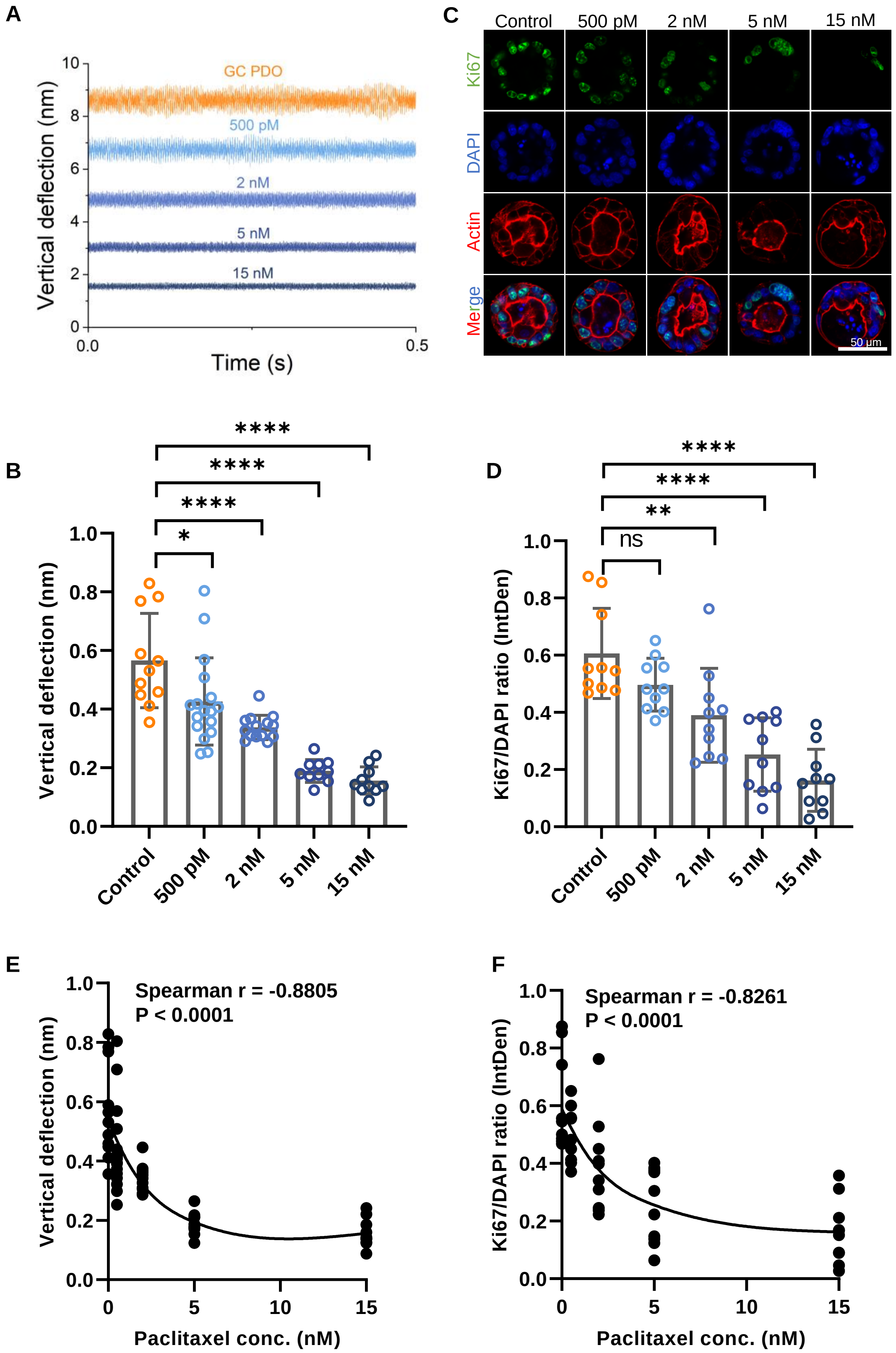
A
C
B
D
E
F
GC PDO
500 pM
2 nM
5 nM
15 nM
Vertical deflection (nm)
Time (s)
Control
Ki67
DAPI
Actin
Merge
50 μm
Ki67/DAPI ratio (IntDen)
ns
Spearman r = -0.8805
P < 0.0001
Spearman r = -0.8261
P < 0.0001
Paclitaxel conc. (nM)

Figure S11 Quantitative correlation data between drug concentration and vibration amplitude or cell proliferation measured on the same set of organoids.

A. Representative raw nanomechanical vibration traces of GC PDOs treated with the indicated concentrations of paclitaxel (500 pM, 2 nM, 5 nM, 15 nM) for 24 h.

B. Statistical analysis of the data shown in Figure S11A. Data are presented as mean ± SD. Statistical significance was assessed using unpaired Student's t-tests. Sample sizes: n = 11 for the control group; n = 17 for the 500 pM paclitaxel group; n = 14 for the 2 nM paclitaxel group; n = 10 for the 5 nM and 15 nM paclitaxel groups.

C. Dose-dependent suppression of proliferation in organoids by paclitaxel. Proliferation was assessed via immunofluorescence staining of Ki67 after 24 hours of drug treatment.

D. Statistical analysis of the data shown in Figure S11C. Data are presented as mean ± SD. Statistical significance was assessed using unpaired Student's t-tests. Sample sizes: n =10 for each group.

E. Spearman correlation between paclitaxel concentration and nanomechanical vibration amplitude. Each point represents the vibration amplitude of a single gastric cancer organoid treated with the indicated concentration of paclitaxel (0, 0.5, 2, 5, 15 nM; total n = 62 organoids). Spearman rank correlation analysis revealed a strong negative monotonic relationship between paclitaxel concentration and vibration amplitude (ρ = -0.8805, 95% CI: –0.9279 to -0.8051, $p < 0.0001$). A smoothing spline curve (knots = 4) was added to visualize the overall trend.

F. Spearman correlation between paclitaxel concentration and Ki67 expression. Each point represents the Ki67/DAPI integrated density ratio of a single gastric cancer organoid treated with the indicated concentration of paclitaxel (0, 0.5, 2, 5, 15 nM; total n = 50 organoids). Spearman rank correlation analysis revealed a strong negative monotonic relationship between paclitaxel concentration and Ki67 expression (ρ = -0.8261, 95% CI: -0.8996 to -0.7072, $p < 0.0001$). A smoothing spline curve (knots = 4) was added to visualize the overall trend.

Figure S12

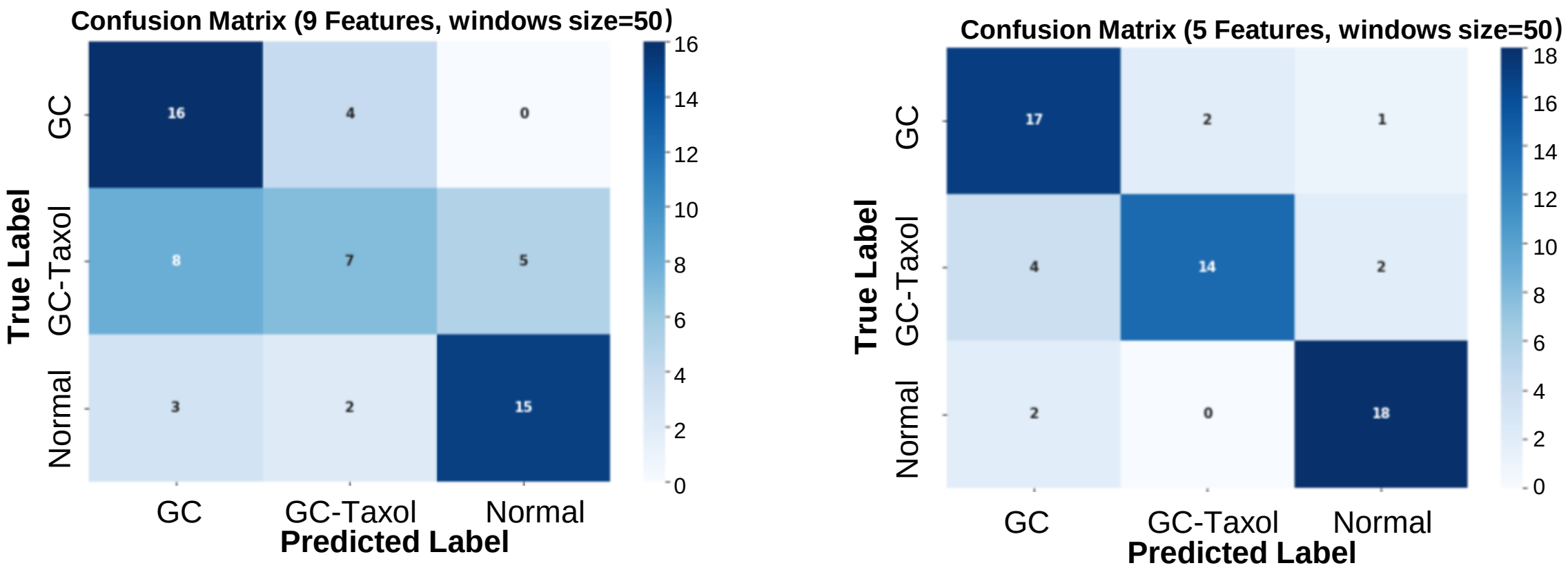

Figure S12 Performance of 1D-CNN classification with varying hyperparameters. The model was evaluated on human normal PDOs, GC PDOs, and paclitaxel-treated GC PDOs using nine features and five features.

Figure S13

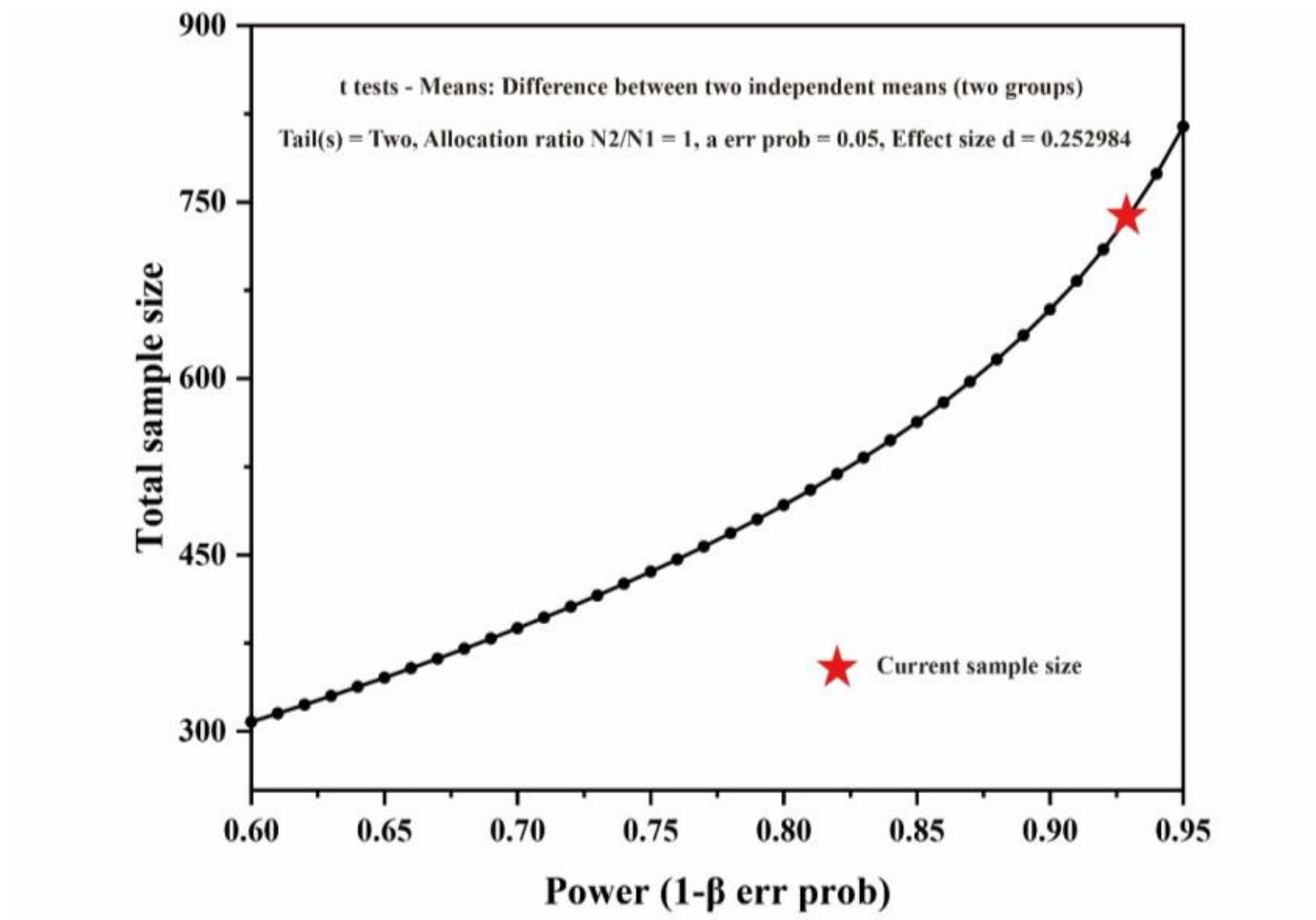

Figure S13 A priori statistical power analysis for the intergroup comparison of machine learning-based measurements between Normal PDO cohort and GC-Taxol-treated PDO cohort.

Figure S14

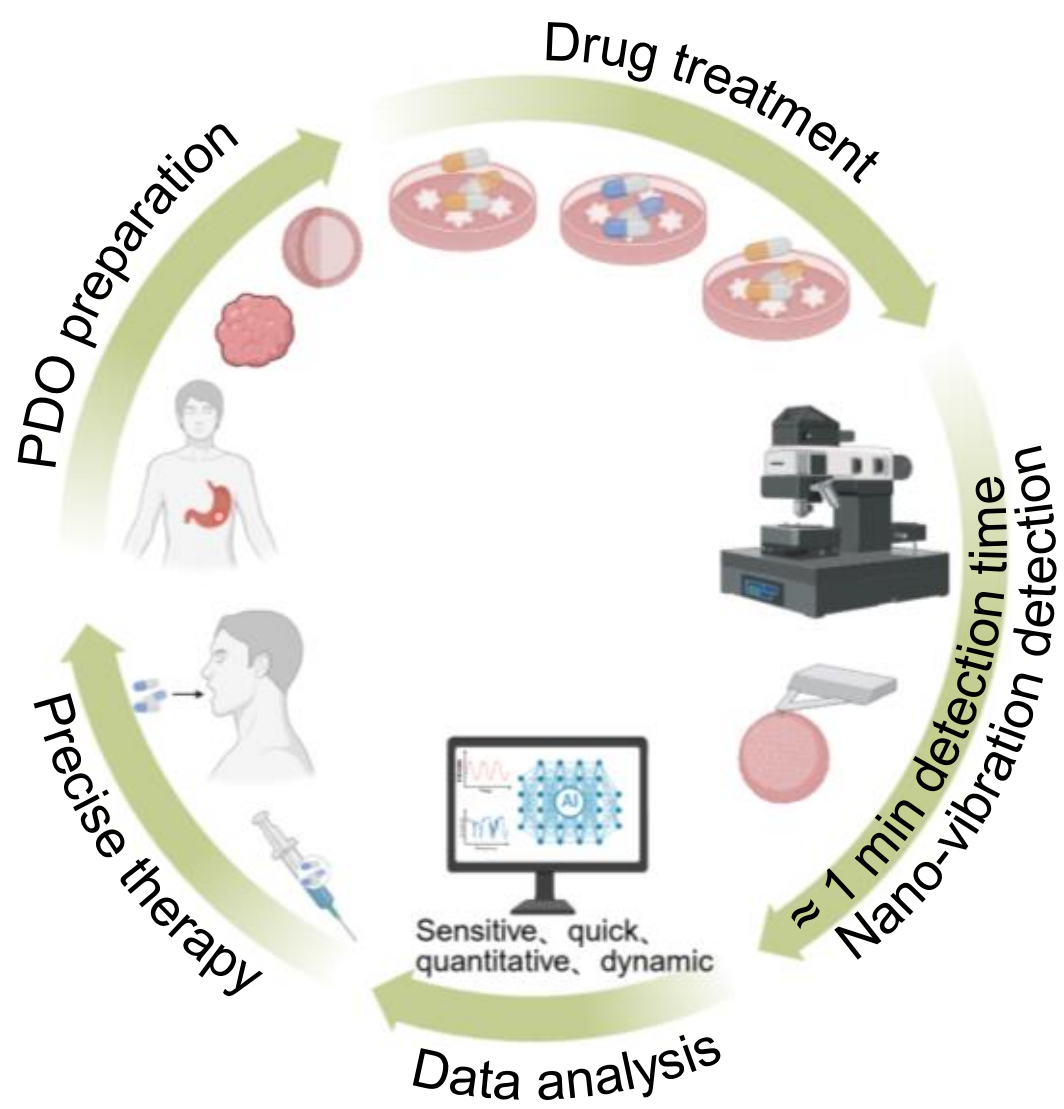

Figure S14 Schematic of the precision medicine model based on nanomechanical vibration profiling of patient-derived organoids (PDOs).

Figure S15

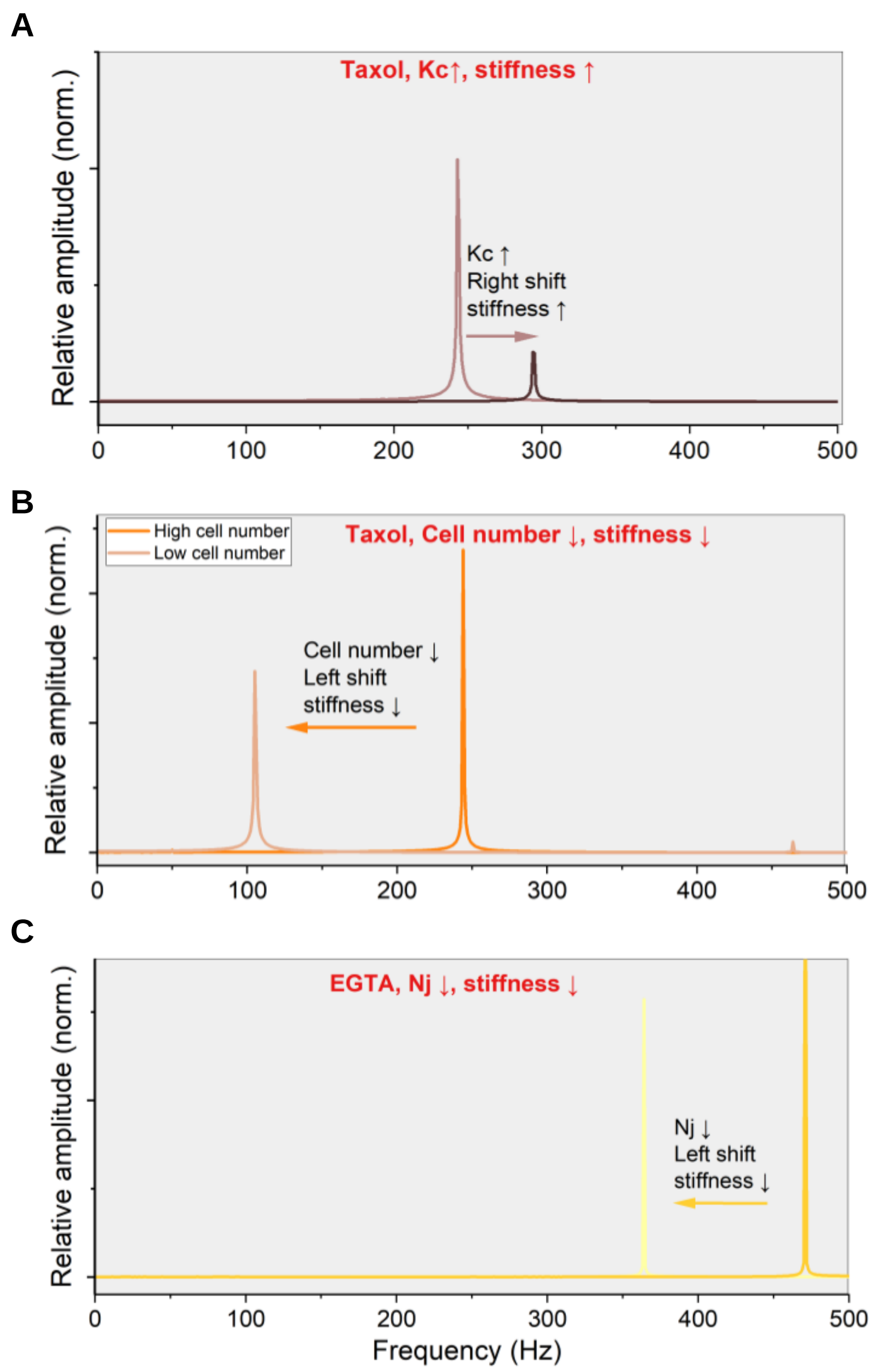

Figure S15 Model prediction of nanomechanical vibration spectrograms for organoids treated with Paclitaxel and EGTA. The observed frequency shifts are used to infer corresponding changes in stiffness.

Supplementary methods

Organoid mechanical model coupling cell viability and structure

The construction of the organoid mechanical model was based on previous literature[1]. In brief, it is difficult to obtain a theoretical solution for organoids using differential equations. Here, we use MATLAB SIMULINK for modeling and numerical simulation. When the organoid structure changes, the parameters of each component in the model are adjusted accordingly. Specifically, k_c is adjusted in response to changes in the number of microtubules, and N_j is adjusted based on changes in the number of intercellular junctions. For changes in cell viability, the modulation is realized by tuning both the amplitude of the excitation force $A = A_0 F(norm.)$ [1] and the total cell number. Cleaved Caspase-3 (Asp175) fluorescence imaging (Figure S8B) confirms that drug treatment leads to a reduction in cell number; therefore, this is used as a direct indicator to characterize changes in cell survival rate in this model.

Deep learning analysis of nanomechanical vibration signals from human gastric cancer organoids

Nanomechanical vibration signals were collected from the experimental groups of patient‑derived normal gastric organoids (Normal PDOs), gastric cancer organoids (GC PDOs) and drug-treated gastric cancer organoids (GC-Taxol PDOs). A total of nine independent patients were enrolled in this study. All signals were recorded at a sampling frequency of 10,000 Hz. For each experimental group, at least four organoids of independent replicate measurements were performed, each acquiring approximately 10 s of vibration data. For deep-learning analysis, only a 0.1 s segment of the 10 s recording is required per organoid replicate. The 0.1 s raw signals were then segmented into non‑overlapping windows of 50 consecutive data points, corresponding to 0.005 s of recording time at 10,000 Hz.

To evaluate the sufficiency of model input windows and enhance the statistical robustness of the analysis, an a priori power analysis was conducted using the standard academic software G*Power 3.1. A conservative estimation strategy

was adopted by selecting the most challenging comparison pair (Normal vs. GC-Taxol, Figure S9D), which exhibited the smallest effect size. Sufficient power to detect this subtle difference ensures the reliability of all other more divergent group comparisons (GC vs. Normal, GC vs. GC-Taxol). The analysis employed an independent samples t-test with a two-tailed design, a significance level (α) of 0.05, and a statistical power (1-β) of 0.9. The Cohen's d effect size was calculated from the standard deviation of 50-point window signal features in the preliminary data of these two groups, aligning with the fundamental input unit of the deep learning model.

To minimize the risk of data leakage and objectively evaluate the model's generalization ability, we strictly followed the standard dataset partitioning protocol in the field of machine learning and adopted a three-part dataset structure: training set, internal validation set, and independent external test set:

1) Training and internal validation datasets: Organoids from 9 independent patients, comprising three experimental groups (Normal, GC, and GC-Taxol), were used for model development. For each patient and each group, at least 4 organoids were measured, and from each organoid, a 0.1-s vibration segment was collected and divided into 20 non-overlapping 50-point windows. Thus, per group, the total number of windows was 9 patients × 4 organoids × 20 windows = 720, all of which were used for analysis. These windows were randomly partitioned into a training set (576 windows, 80%) and an internal validation set (144 windows, 20%).

2) Independent external test set: We separately collected extra 3 experimental groups (Test_Normal, Test_GC, Test_GC-Taxol) that were completely independent of the training and internal validation datasets and kept them fully isolated throughout the entire model training process. The 97% classification accuracy reported in Figure 7F of the manuscript is precisely the evaluation result based on this independent external test set, rather than the performance on the training set or internal validation set.

Table S1 1D-CNN model parameters applied

Name	Filters	Kernel size/stride	Units	Input size	Output size	Activation function	No. of trainable parameters
**Conv_1**	32	3/1	-	5×1	32×5	relu	128
**MaxPooling_1**	-	2/2	-	32×5	32×2	-	0
**Conv_2**	16	3/1	-	32×2	16×2	relu	1552
**MaxPooling_2**	-	2/2	-	16×2	16×1	-	0
**Dense_1**	-	-	32	1×16	1×32	relu	544
**Dense_2**	-	-	4	1×32	1×4	softmax	132
						Total	2356

Table S2 Organoid mechanical model parameters

Parameters	Meaning	Value	Source
$\boldsymbol{S_c}$	Organoid surface area	8000 μm^2	Calculated from Fig.S8A
$\boldsymbol{h_c}$	Organoid height	100 μm	Calculated from Fig.S8A
$\boldsymbol{\rho_c}$	Cell density	1.1×10^3 kg/m^3	Reference[2]
$\boldsymbol{k_c}$	Cell stiffness of each part	4.5 pN/nm	Calculated from the data in Reference[3]
$\boldsymbol{k_j}$	Cell–Cell junction stiffness	0.4 pN/nm	Fitted by the figure in Reference[4]
$\boldsymbol{k_a}$	Fibronectin stiffness	5 pN/nm	Reference[5]
$\boldsymbol{N_j}$	Cell–Cell junction number of each part	220	This study
$\boldsymbol{A_0}$	Initial amplitude of a single cell	1×10^{-9} nm	This study
$\boldsymbol{N_a}$	Fibronectin molecule number of organoids	6000	Calculated from the data in Reference[5]
$\boldsymbol{k_m}$	Microcantilever stiffness	0.12 N/m	Product description
$\boldsymbol{m_m}$	Microcantilever equivalent mass	6×10^{-9} g	Calculated from product description

Reference

[1] W. Wu, Y. Peng, M. Xu, T. Yan, D. Zhang, Y. Chen, K. Mei, Q. Chen, X. Wang, Z. Qiao, C. Wang, S. Wu, Q. Zhang, *ACS nano* **2024**, *18* (4), 3480, https://doi.org/10.1021/acsnano.3c10811.

[2] W. H. Grover, A. K. Bryan, M. Diez-Silva, S. Suresh, J. M. Higgins, S. R. Manalis, *Proceedings of the National Academy of Sciences of the United States of America* **2011**, *108* (27), 10992, https://doi.org/10.1073/pnas.1104651108.

[3] M. Lekka, *Bionanoscience* **2016**, *6*, 65, https://doi.org/10.1007/s12668-016-0191-3.
[4] O. Shafraz, M. Rübsam, S. N. Stahley, A. L. Caldara, A. P. Kowalczyk, C. M. Niessen, S. Sivasankar, *eLife* **2018**, *7*, https://doi.org/10.7554/eLife.37629.
[5] Z. Gong, S. E. Szczesny, S. R. Caliari, E. E. Charrier, O. Chaudhuri, X. Cao, Y. Lin, R. L. Mauck, P. A. Janmey, J. A. Burdick, V. B. Shenoy, *Proceedings of the National Academy of Sciences of the United States of America* **2018**, *115* (12), E2686, https://doi.org/10.1073/pnas.1716620115.
